# Assessing the efficacy of immunotherapy in lung squamous carcinoma using artificial intelligence neural network

**DOI:** 10.3389/fimmu.2022.1024707

**Published:** 2022-11-28

**Authors:** Siqi Li, Wei Li, Tianyu Ma, Siyun Fu, Xiang Gao, Na Qin, Yuhua Wu, Xinyong Zhang, Jinghui Wang, Yuanming Pan, Zhidong Liu

**Affiliations:** ^1^ Department of Thoracic Surgery, Beijing Chest Hospital/Beijing Tuberculosis and Thoracic Tumor Research Institute, Capital Medical University, Beijing, China; ^2^ Department of Medical Oncology, Beijing Tuberculosis and Thoracic Tumor Research Institute/Beijing Chest Hospital, Capital Medical University, Beijing, China; ^3^ Cancer Research Center, Beijing Tuberculosis and Thoracic Tumor Research Institute/Beijing Chest Hospital, Capital Medical University, Beijing, China

**Keywords:** immunotherapy, lung squamous carcinoma, neural network, deep learning, predictive model

## Abstract

**Background:**

At present, immunotherapy is a very promising treatment method for lung cancer patients, while the factors affecting response are still controversial. It is crucial to predict the efficacy of lung squamous carcinoma patients who received immunotherapy.

**Methods:**

In our retrospective study, we enrolled lung squamous carcinoma patients who received immunotherapy at Beijing Chest Hospital from January 2017 to November 2021. All patients were grouped into two cohorts randomly, the training cohort (80% of the total) and the test cohort (20% of the total). The training cohort was used to build neural network models to assess the efficacy and outcome of immunotherapy in lung squamous carcinoma based on clinical information. The main outcome was the disease control rate (DCR), and then the secondary outcomes were objective response rate (ORR), progression-free survival (PFS), and overall survival (OS).

**Results:**

A total of 289 patients were included in this study. The DCR model had area under the receiver operating characteristic curve (AUC) value of 0.9526 (95%CI, 0.9088–0.9879) in internal validation and 0.9491 (95%CI, 0.8704–1.0000) in external validation. The ORR model had AUC of 0.8030 (95%CI, 0.7437–0.8545) in internal validation and 0.7040 (95%CI, 0.5457–0.8379) in external validation. The PFS model had AUC of 0.8531 (95%CI, 0.8024–0.8975) in internal validation and 0.7602 (95%CI, 0.6236–0.8733) in external validation. The OS model had AUC of 0.8006 (95%CI, 0.7995–0.8017) in internal validation and 0.7382 (95%CI, 0.7366–0.7398) in external validation.

**Conclusions:**

The neural network models show benefits in the efficacy evaluation of immunotherapy to lung squamous carcinoma patients, especially the DCR and ORR models. In our retrospective study, we found that neoadjuvant and adjuvant immunotherapy may bring greater efficacy benefits to patients.

## Highlights

- The expression level of PD-L1 is not an ideal tool for evaluating efficacy.
- The neural network models show benefits in the efficacy evaluation of immunotherapy to lung squamous carcinoma patients, especially the DCR and ORR models.
- Neoadjuvant and adjuvant immunotherapy may bring greater efficacy benefits to lung squamous carcinoma patients.

## Background

Lung cancer occupies first place in mortality and second place in morbidity globally (1, 2). Non-small cell lung cancer (NSCLC) accounts for about 85% of lung cancer cases, and the 5-year survival rate is only 18% (3, 4). Recently, the advent of targeted drugs and the emergence of immunotherapy have significantly prolonged the survival of lung cancer patients and improved their quality of life (3). Lung squamous carcinoma accounts for 25%–30% of lung cancer cases, while the occurrence rate of common driver gene mutation is less than 7% (5–9). Patients with lung squamous carcinoma have few chances to receive targeted therapy. Fortunately, immunotherapy brings a new light to patients with lung squamous carcinoma, which can significantly improve objective response rate (ORR) and prolong progression-free survival (PFS) and overall survival (OS) (10–12). However, it also brings challenges to the selection of biomarkers for predicting efficacy, the treatment plan for lung cancer, and treatment-related adverse events.

At present, the expression level of programmed cell death ligand 1 (PD-L1) is an indicator that may predict the effectiveness of anti-programmed cell death 1 (anti-PD-1)/PD-L1 immunotherapy and screen the population sensitive to them (13). However, studies also found that some patients with high PD-L1 expression have a poor immune response. On the contrary, up to 10% of patients with negative PD-L1 expression have a good immune response (14). It suggested that the expression level of PD-L1 is not an ideal tool for evaluating efficacy. Several studies showed that traditional clinical or pathological features, including smoking status, age, pathological type, and tumor grade, are associated with immunotherapy for NSCLC (15–17). Therefore, it is necessary to use a new method to evaluate the efficacy of immunotherapy and identify the dominant population sensitive to immunotherapy.

Deep learning neural network, as a subdiscipline of artificial intelligence (AI), has shown good performance in predicting and monitoring treatment response, which is also gradually gaining the attention of clinicians (18, 19). Convolutional neural network is currently used to diagnose solid tumors (lung cancer, melanoma, gastrointestinal tumors, etc.) through automatic quantification of radiological images, digital histopathological image interpretation, or biomarker analysis (18, 20–22). However, little research focused on the evaluation of immunotherapy efficacy in NSCLC based on AI.

To better predict the efficacy of the immunotherapy of lung squamous carcinoma patients and thus further provide more optimal treatment strategies, we introduced the neural network algorithm to build a fully connected neural network (also known as a dense neural network (DNN)) based on clinical information of the above patients. The original codes and data have been uploaded for use by clinicians and future visualization platforms.

## Method

### Study design and clinical information

**Figure 1:**
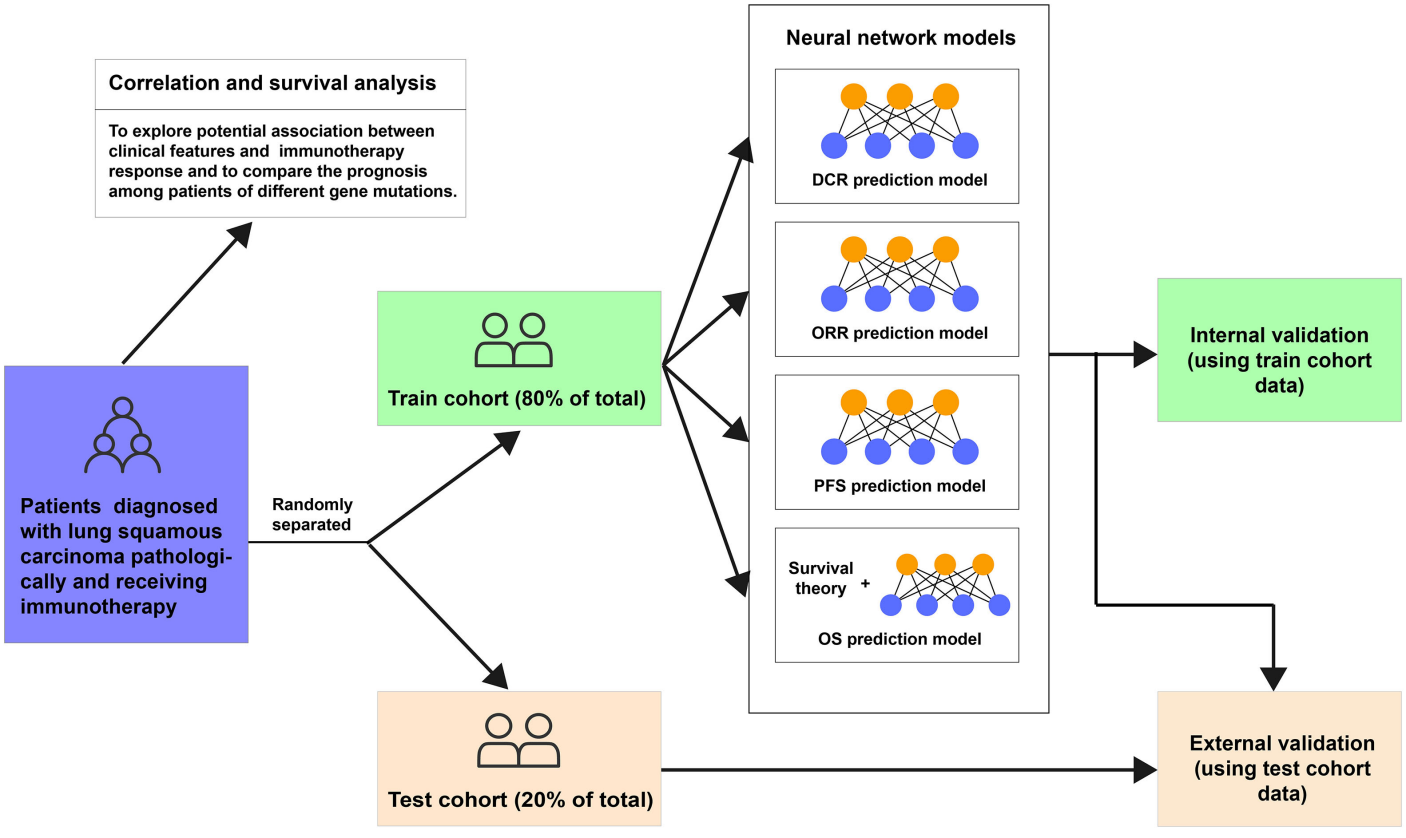
Flowchart of this study. DCR, disease control rate; ORR, objective response rate; PFS, progression-free survival; OS, overall survival.

This study was designed as a retrospective cohort study. Eligible patients aged ≥18 years were diagnosed with lung squamous carcinoma pathologically and received immunotherapy in Beijing Chest Hospital affiliated with Capital Medical University between 16 January 2017 and 10 December 2021. Patients with active autoimmune disease, symptomatic interstitial lung disease, multiple primary pulmonary carcinomas, or missing any of the included clinical characteristics, like status or follow-up records, were excluded. Tumor PD-L1 expression was assessed using the PD-L1 immunohistochemistry 22C3 pharmDx kit (Agilent Technologies, Carpinteria, CA, USA) at the Pathology Department of Beijing Chest Hospital. The expression level of PD-L1 protein of archival tumor tissue or tissue obtained through biopsy was determined by the tumor proportion score (TPS). Then, all patients were grouped into two cohorts randomly, the training cohort (80% of the total) and the test cohort (20% of the total). The training cohort was used to build neural network models to assess the efficacy and outcome of immunotherapy in lung squamous carcinoma based on clinical information, with evaluated by internal validation (using training cohort data) and external validation (using test cohort data) (**Figure 1**). The last follow-up date was 30 April 2022.

This research has been approved by the Ethics Committee of Beijing Chest Hospital affiliated with Capital Medical University. Given that this was a retrospective analysis, individual consent was waived.

### Potential predictive variables

The potential clinical predictive variables were as follows: age, sex, smoking status, performance status (PS) score before receiving immunotherapy, PD-L1 expression, TNM and clinical stage, vascular invasion, pleural metastasis, extra-thoracic metastasis, brain metastasis, liver metastasis, bone metastasis, adrenal metastasis, received chemoimmunotherapy or not, received immunotherapy plus antiangiogenic therapy or not, neoadjuvant immunotherapy, immunotherapy lines, and gene mutations. Considering that gene mutations contained much missing value, we did not include them in the final models. **Supplementary Figure 1** shows the distribution of missing values. TNM and clinical stage were evaluated by at least two senior clinicians, referring to the 8th edition of the American Joint Committee on Cancer (AJCC) stage. All patients have received immunotherapy, of whom some accepted another therapy such as chemotherapy or antiangiogenic medicine. Partial patients in this study were treated with neoadjuvant immunotherapy, which meant they would undergo operations later.

Multiple imputations of missing values have been performed before the above predictive variables were included in models, with the help of the R package (23).

### Outcome

The main outcome was the disease control rate (DCR), and secondary outcome indicators were ORR, PFS, and OS. The imaging manifestations have been interpreted by local researchers according to the Response Evaluation Criteria in Solid Tumors (RECIST version 1.1). The best overall response (BOR) was assessed including complete response (CR), partial response (PR), or stable disease (SD) after immunotherapy, regarded as DCR. ORR included CR or PR patients. PFS was defined as the time from the day of receiving immunotherapy to objective tumor progression, surgery, or death. We processed PFS as a binary variable, PFS ≤6 months or >6 months.

### Data pre-processing

Standardization of data is a common process in many machine learning situations, which implies numerical variables subtracting their means and dividing by their standard deviations. Categorical variables have been converted into dummy variables, such as replacing the sex variable with two dummy variables (female = 0 or male = 1). Age and PS score before receiving immunotherapy were standardized, while sex, smoking status, PD-L1 expression, TNM and clinical stage, vascular invasion, pleural metastasis, extra-thoracic metastasis, brain metastasis, liver metastasis, bone metastasis, adrenal metastasis, received chemoimmunotherapy or not, received immunotherapy plus antiangiogenic therapy or not, neoadjuvant immunotherapy, and immunotherapy lines were transformed to dummy variables. Data from the test cohort were standardized according to the training cohort, and **Supplementary Table 1** exhibits the mean and standard deviations of numerical variables.

Python package pandas and scikit-learn helped us to achieve the above processing (24, 25).

### Model training and validation

To assess the DCR, ORR, and PFS, three dense neural networks have been built. To obtain accurate predictions, batch training and normalization were used. Dropout layers were used to avoid overfitting (which means performing well in the training cohort but badly in the test cohort), and early stopping functions were used to end training epochs if necessary.

To predict the OS possibility of lung squamous carcinoma patients after immunotherapy, we conducted a neural network survival model based on Katzman’s DeepSurv (26). It has to be mentioned that the neural network was designed to tackle traditional classification issues instead of time-dependent tasks, so the performance of OS prediction might be moderate.

As mentioned above, since our models were applied to solve classification problems, we used the area under the receiver operating characteristic curve (AUC) to evaluate their performance. The closer the AUC is to 1, the better the performance of the model. In order to obtain AUC with detailed 95% confidence interval (CI), we run bootstrap 1,000 times.

Python and its packages PyTorch, torchtuples, NumPy, pycox, and matplotlib and the R package pROC helped us in these analyses (27–32).

### Correlation and survival analysis

We used correlation analysis to explore the potential association between clinical features and patients’ immunotherapy response with Spearman’s rank correlation, visualized by heatmap and chord diagram. Finally, survival analysis was performed to compare the prognoses of patients with different gene mutations. R and packages corrplot, circlize, and survminer were used during these procedures (33–35).

### Statistical analysis

The numerical data of skewed distribution were analyzed by the Wilcoxon test, while categorical data were compared by chi-square or Fisher’s exact test. A two-sided p-value of less than 0.05 was considered statistically significant. These analyses and relevant plotting were completed using R software and packages epiDisplay, ggplot2, and ggridges (36, 37).

## Result

### Characteristics of patients

**Figure 2:**
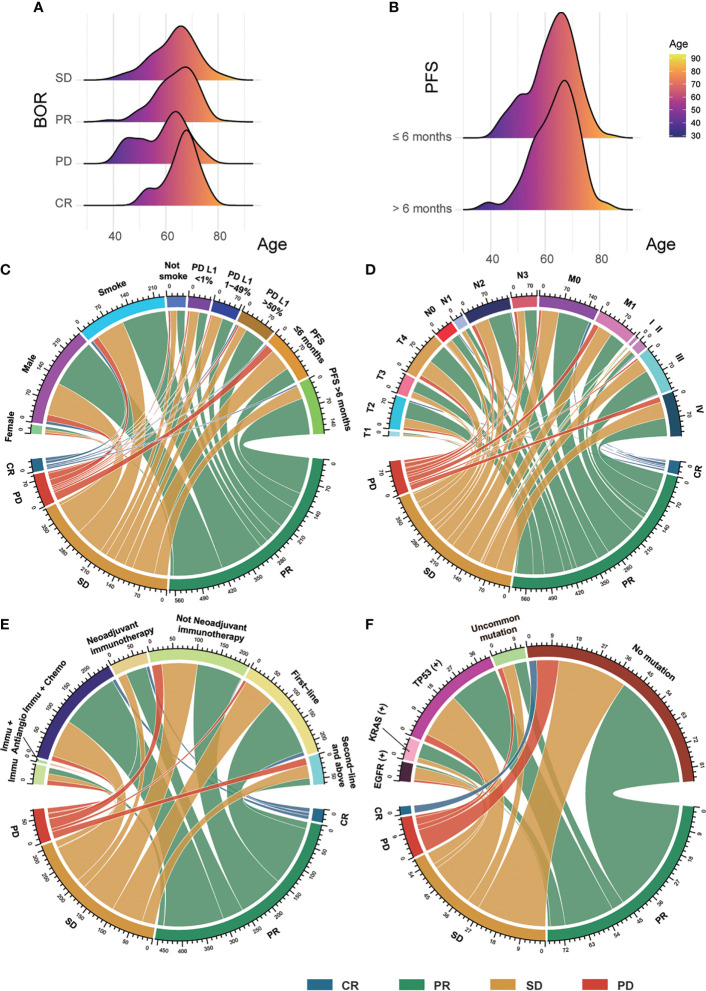
The clinical features of patients. **(A)** The distribution between BOR and patients’ age. **(B)** The distribution between PFS and patients’ age. **(C)** The connections among patients’ sex, smoking status, PD-L1 expression, and PFS with their BOR. **(D)** The connections among patients’ TNM stage with their BOR. **(E)** The correlations between patients’ therapy and their BOR. **(F)** The correlations between patients’ gene mutation and their BOR. BOR, best overall response; CR, complete response; PR, partial response; SD, stable disease; PD, progressive disease; PFS, progression-free survival; PD-L1, programmed cell death ligand 1; Immu, immunotherapy; Antiangio, antiangiogenic therapy; Chemo, chemotherapy; uncommon gene mutation.

A total of 289 patients were included; the PFS of 138 patients was shorter or equal to 6 months, and the PFS of 151 patients was longer than 6 months. In the group with PFS ≤ 6 months, there were 11 women and 127 men. As for the expression of PD-L1, 30 patients had <1% expression, 37 patients had 1%–49% expression, and 42 patients had ≥50% expression. Among them, four patients had CR, 44 patients had PR, 65 patients had SD, and 24 patients were diagnosed with PD. The group with PFS > 6 months included 12 women and 139 men. The PD-L1 expression of 29 patients was <1%; 36 patients, between 1% and 49%; and 55 patients, ≥50%. Among them, five had CR, 108 had PR, and 38 had PD. The median follow-up time was 7.75 months in the group with PFS ≤ 6 months and 12.8 months in the group with PFS > 6 months. The detailed information of patients is illustrated in **Table 1** and **Figure 2**.

**Table 1:** The clinical features of patients.

	PFS		Statistical method	p-Value
	≤6 months	>6 months
	(N = 138)	(N = 151)
	N (%)		
Age			Wilcoxon	0.3461
Median (IQR)	64 (58, 68.75)	65 (58.5, 69)		
Sex			Chi-square	0.9940
Female	11 (7.97)	12 (7.95)		
Male	127 (92.03)	139 (92.05)		
Smoke			Chi-square	0.3291
No	27 (19.57)	23 (15.23)		
Yes	110 (79.71)	127 (84.11)		
Unknown	1 (0.72)	1 (0.66)		
PS score			Wilcoxon	0.0435*
Median (IQR)	1 (1,1)	1 (1,1)		
PD-L1 expression			Chi-square	0.5360
<1%	30 (21.74)	29 (19.21)		
1%–49%	37 (26.81)	36 (23.84)		
>50%	42 (30.43)	55 (36.42)		
Unknown	29 (21.01)	31 (20.53)		
T			Fisher’s exact	0.1763
1	3 (2.17)	2 (1.32)		
1b	3 (2.17)	0 (0)		
1c	4 (2.90)	1 (0.66)		
2	22 (15.94)	34 (22.52)		
2a	7 (5.07)	11 (7.28)		
2b	7 (5.07)	12 (7.95)		
3	25 (18.12)	32 (21.19)		
3b	1 (0.72)	0 (0)		
4	66 (47.83)	59 (39.07)		
N			Chi-square	0.1234
0	19 (13.77)	34 (22.52)		
1	11 (7.97)	18 (11.92)		
2	68 (49.28)	63 (41.72)		
3	37 (26.81)	33 (21.85)		
Unknown	3 (2.17)	3 (1.99)		
M			Chi-square	0.0261*
0	69 (50.00)	98 (64.90)		
1	7 (5.07)	3 (1.99)		
1a	19 (13.77)	22 (14.57)		
1b	12 (8.70)	5 (3.31)		
1c	27 (19.57)	18 (11.92)		
Unknown	4 (2.90)	5 (3.31)		
Clinical stage			Fisher’s exact	0.0335*
IA	2 (1.45)	0 (0)		
IB	1 (0.72)	5 (3.31)		
IIA	4 (2.90)	8 (5.30)		
IIB	3 (2.17)	11 (7.28)		
IIIA	25 (18.12)	36 (23.84)		
IIIB	29 (21.01)	30 (19.87)		
IIIC	4 (2.90)	7 (4.64)		
IV	70 (50.72)	54 (35.76)		
Vascular invasion			Chi-square	0.9061
No	132 (95.65)	144 (95.36)		
Yes	6 (4.35)	7 (4.64)		
Pleural metastasis			Chi-square	0.0169*
No	113 (81.88)	138 (91.39)		
Yes	25 (18.12)	13 (8.61)		
Extra-thoracic metastasis			Chi-square	0.0052**
No	96 (69.57)	126 (83.44)		
Yes	42 (30.43)	25 (16.56)		
Brain metastasis			Chi-square	0.0192*
No	124 (89.86)	146 (96.69)		
Yes	14 (10.14)	5 (3.31)		
Liver metastasis			Chi-square	0.6595
No	128 (92.75)	142 (94.04)		
Yes	10 (7.25)	9 (5.96)		
Bone metastasis			Chi-square	0.0088**
No	109 (78.99)	136 (90.07)		
Yes	29 (21.01)	15 (9.93)		
Adrenal metastasis			Chi-square	0.2243
No	128 (92.75)	145 (96.03)		
Yes	10 (7.25)	6 (3.97)		
Gene mutation			Fisher’s exact	0.1202
EGFR	5 (3.62)	2 (1.32)		
KRAS	1 (0.72)	7 (4.64)		
TP53	15 (10.87)	26 (17.22)		
Uncommon	7 (5.07)	5 (3.31)		
Negative	40 (28.99)	47 (31.13)		
Unknown	70 (50.72)	64 (42.38)		
Therapy			Fisher’s exact	0.4352
Immu	23 (16.67)	18 (11.92)		
Immu + Antiangio	3 (2.17)	2 (1.32)		
Immu + Chemo	112 (81.16)	131 (86.75)		
Neoadjuvant immunotherapy			Chi-square	<0.001***
No	115 (83.33)	97 (64.24)		
Yes	23 (16.67)	54 (35.76)		
Immunotherapy lines			Chi-square	<0.001***
First-line	94 (68.12)	131 (86.75)		
Second-line and above	44 (31.88)	20 (13.25)		
BOR			Fisher’s exact	<0.001***
CR	4 (2.90)	5 (3.31)		
PR	44 (31.88)	108 (71.52)		
SD	65 (47.10)	38 (25.17)		
PD	24 (17.39)	0 (0)		
Unknown	1 (0.72)	0 (0)		
ORR			Chi-square	<0.001***
No	89 (64.49)	38 (25.17)		
Yes	48 (34.78)	113 (74.83)		
Unknown	1 (0.72)	0 (0)		
DCR			Chi-square	<0.001***
No	24 (17.39)	0 (0)		
Yes	113 (81.88)	151 (100.00)		
Unknown	1 (0.72)	0 (0)		
PFS			Wilcoxon	<0.001***
Median (IQR)	3.26 (2, 4.55)	10.07 (7.68, 13.33)		
OS			Wilcoxon	<0.001***
Median (IQR)	7.75 (4.45, 13.2)	12.8 (10.67, 18.57)		

PFS, progression-free survival; IQR, interquartile range; PS score, performance status score; PD-L1, programmed cell death ligand 1; Immu, immunotherapy; Antiangio, antiangiogenic therapy; Chemo, chemotherapy; BOR, best overall response; CR, complete response; PR, partial response; SD, stable disease; PD, progressive disease; ORR, objective response rate; DCR, disease control rate; OS, overall survival.

* P<0.05, ** P<0.01, ***P<0.001.

### Model training

We conducted four neural networks to assess the efficacy and outcome of immunotherapy in lung squamous carcinoma (**Figure 1**). The DCR model was designed to return the probabilities of patients showing DCR after immunotherapy, with the ORR model predicting the ORR possibilities and the PFS model judging their PFS longer or shorter than 6 months. The OS model was used to predict patients’ OS possibility based on a neural network survival algorithm. The training curves are shown in **Supplementary Figure 2**.

### Model performance and inference

**Figure 3:**
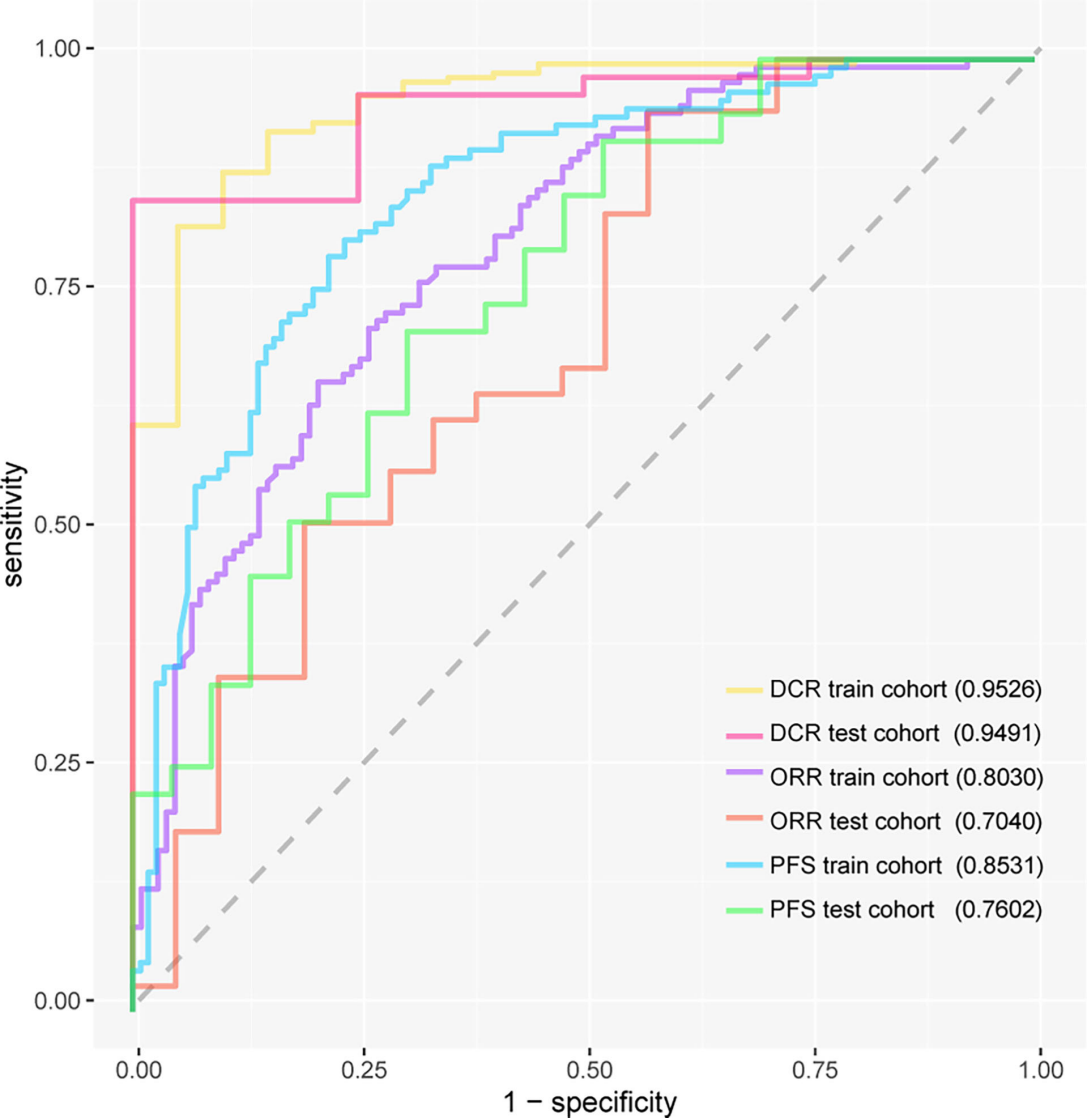
The receiver operating characteristic curves of three models. DCR, disease control rate; ORR, objective response rate; PFS, progression-free survival.

The DCR model had AUC of 0.9526 (95%CI, 0.9088–0.9879) in internal validation and 0.9491 (95%CI, 0.8704–1.0000) in external validation. The ORR model had AUC of 0.8030 (95%CI, 0.7437–0.8545) in internal validation and 0.7040 (95%CI, 0.5457–0.8379) in external validation. The PFS model had AUC of 0.8531 (95%CI, 0.8024–0.8975) in internal validation and 0.7602 (95%CI, 0.6236–0.8733) in external validation. The receiver operating characteristic curve valuess of the DCR model, ORR model, and PFS model are shown in **Figure 3**, and their original codes are shown in **Supplementary Material File 1**. The OS model had AUC of 0.8006 (95%CI, 0.7995–0.8017) in internal validation and 0.7382 (95%CI, 0.7366–0.7398) in external validation (**Table 2**). The codes of the OS model are shown in **Supplementary Material File 2**. The weights and hyper-parameters of the above four models are shown in **Supplementary Material File 3**.

**Table 2:** The performance of OS model.

	AUC	95%CI
Training cohort	0.8006	0.7995–0.8017
Test cohort	0.7382	0.7366–0.7398

OS, overall survival; AUC, area under the receiver operating characteristic curve; CI, confidence interval.

Age, sex, smoking status, PS score, PD-L1 expression, TNM and clinical stage, vascular invasion, pleural metastasis, extra-thoracic metastasis, brain metastasis, liver metastasis, bone metastasis, adrenal metastasis, received chemoimmunotherapy or not, received immunotherapy plus antiangiogenic therapy or not, neoadjuvant immunotherapy, and immunotherapy lines were the predictive clinical features. When these codes are open in a python environment (https://www.python.org/) and jupyter notebook software (https://www.jupyter.org/), with predictive clinical features inputted, models will return the predictive possibility of DCR, ORR, PFS, or OS.

### Correlation analysis

**Figure 4:**
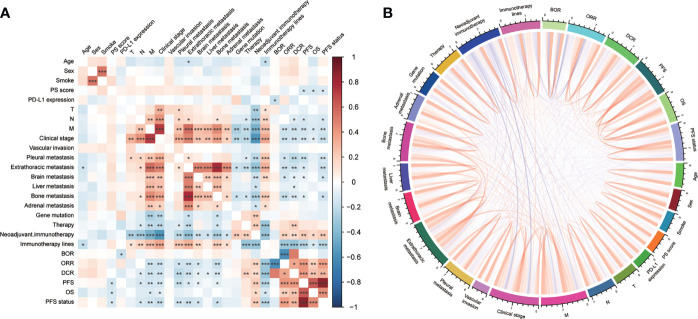
The correlation among clinical features with immunotherapy was visualized by **(A)** heatmap and **(B)** chord diagram. PD-L1, programmed cell death ligand 1; BOR, best overall response; ORR, objective response rate; DCR, disease control rate; PFS, progression-free survival; OS, overall survival. *P<0.05, **P<0.01, ***P<0.001.

General overview, PS score, PD-L1 expression, TNM stage, distant metastasis, and invasion (vascular, pleural, brain, liver, and bone) had statistical relation with immunotherapy (BOR, DCR, ORR, PFS, or OS) (**Figure 4**). We also compared the OS of patients with different gene mutations but did not find some statistical discrepancies (**Supplementary Figure 3**).

## Discussion

Immunotherapy significantly improves the prognosis of lung cancer patients, but not everyone who receives immunotherapy can benefit from it (38). Hence, it is essential to choose a better method to predict immunotherapy efficacy and screen the population sensitive to immunotherapy. We developed and validated deep learning neural network models based on clinical data for immune efficacy prediction for lung squamous carcinoma. The results of our study verified that the deep learning model showed good predictive performance in these patients.

Currently, multiple clinical trials have shown that the expression level of PD-L1 not only could decide who to treat but also could hint at whom to benefit (16, 39–42). Interestingly, several trials have examined PD-L1 as a viable biomarker to predict response to immune checkpoint inhibitors (ICIs) (43–45). Moreover, due to the heterogeneity of PD-L1 expression in tumors, there is a certain difference between puncture biopsy specimens and surgical resection specimens (46, 47). Some studies have shown that the positive rate of PD-L1 was related to clonal selection, biopsy sites, and detection time, and researchers should further coordinate the harmonization of utilized clones, scores, and interobserver variability (48). It is suggested that the independent predictive effect of tumor PD-L1 expression is still imperfect.

We developed and validated the immunotherapy predictive deep learning models using clinical information in lung squamous carcinoma. Raw data were divided into two independent groups, the training cohort (80% of the total) and the test cohort (20% of the total). Age, sex, smoking status, PS score before receiving immunotherapy, PD-L1 expression, TNM and clinical stage, vascular invasion, pleural metastasis, extra-thoracic metastasis, brain metastasis, liver metastasis, bone metastasis, adrenal metastasis, received chemoimmunotherapy or not, received immunotherapy plus antiangiogenic therapy or not, neoadjuvant immunotherapy, and immunotherapy lines were chosen as the predictive variables. The train cohort was used to conduct the ORR model, DCR model, PFS model, and OS model, which were validated using both train cohort and test cohort. Teo avoid overfitting, early stopping function and dropout layers were adopted after numerical variables were standardized and categorical variables were converted into dummy variables. Finally, the above models showed satisfactory performances. The DCR model had AUC of 0.9526 (95%CI, 0.9088–0.9879) in internal validation and 0.9491 (95%CI, 0.8704–1.0000) in external validation. The ORR model had AUC of 0.8030 (95%CI, 0.7437–0.8545) in internal validation and 0.7040 (95%CI, 0.5457–0.8379) in external validation. The PFS model had AUC of 0.8531 (95%CI, 0.8024–0.8975) in internal validation and 0.7602 (95%CI, 0.6236–0.8733) in external validation. The benefit of immunotherapy can be predicted by deep learning models that integrate patient clinical information. Compared with PD-L1 expression, the efficacy indicators DCR and ORR predicted it more accurately.

As a new efficacy prediction model, the deep learning model will have the potential to support clinical decision-making more accurately. She et al. evaluated the use of deep learning algorithms to evaluate the specific survival of NSCLC patients and concluded that deep learning was significantly better than previous models in lung cancer prognosis assessment and treatment recommendations (18). Mu et al. used PET/CT image deep learning to measure the PD-L1 state to predict immunotherapy response non-invasively and then found that deep learning can replace PD-L1 detected by immunohistochemistry (IHC) (49). In our study, we enrolled immunotherapy patients with lung squamous carcinoma, including stage I–IV, for model training and testing. The deep learning models have multiple hidden layers, each of which contains multiple nodes. The node weights between different layers are updated in time according to the loss function and the reverse propagation of the optimizer. Coupled with the application of activation functions, deep learning can better learn and simulate the non-linear relationship between predictive variables and outcomes than traditional statistical models and some machine learning algorithms. Realistic data, especially clinical data, have a relationship that is not simple linear but intricate. Deep learning is more suitable for the analysis and modeling of clinical data. Therefore, our research found that this will be an interesting attempt, and the models also showed satisfactory performance and could precisely predict the efficacy of immunotherapy.

Of note, ICIs have contributed to improving the survival of patients with lung squamous carcinoma. Our results found that first-line immunotherapy can increase DCR to 86.7% and ORR to 46.7%, which is similar to other clinical studies. In KEYNOTE-024 (50), pembrolizumab, compared with chemotherapy, can remarkably improve the ORR (44.8% *vs.* 27.8%).

With the development of immunotherapy, many studies have found that chemo-immunotherapy strategy can significantly improve the response of NSCLC patients (51–55). The KEYNOTE-407 (53) study found that first-line immunotherapy combined with chemotherapy was better than chemotherapy; the ORR was 66.6% versus 38.4%, respectively. A plethora of phase III clinical trials such as IMpower110 (54), CameL-Sq (52), ORIT-12 (51), and GEMSTONE-302 (55) showed superior efficacy with ICIs plus chemotherapy compared with chemotherapy alone. In our study, we observed that the DCR of patients who received chemoimmunotherapy was 97.7% and ORR was 62.93%, which were similar to the above studies. To be brief, first-line immunotherapy combined with chemotherapy has rapidly expanded first-line treatment options for advanced NSCLC patients without sensitizing epidermal growth factor receptor (EGFR) mutations or anaplastic lymphoma kinase (ALK) fusions.

The success of ICIs in NSCLC has expanded to unresectable stage III and more recently to resectable stage II–IIIA disease. The NADIM study supported the addition of neoadjuvant nivolumab to platinum-based chemotherapy in patients with resectable stage IIIA NSCLC; the major pathological response (MPR) rate was 83%, pathological complete response (pCR) rate was 71%, and 90% of patients had tumor stage decline (33 cases) (56). In CheckMate 816, the neoadjuvant treatment of nivolumab plus chemotherapy improved the pCR rate (24.0% *vs.* 2.2%) in resectable NSCLC patients, and median OS showed a beneficial trend (57). In a neoadjuvant study of sintilimab combined with chemotherapy, 40.5% and 10.8% of patients attained MPR and pCR, respectively, and 3-year OS and disease-free survival (DFS) rates were 95.5% and 81.8%, respectively (58). In our study, we found that the MPR rate of patients with neoadjuvant immunotherapy was 58.4% and the pCR rate was 38.9%, which was similar to the above studies. Neoadjuvant chemoimmunotherapy could change the perception of locally advanced lung cancer from being a potentially lethal disease to one that is curable.

In summary, it is found that immunotherapy can improve the efficacy of patients to a certain extent, but the factors affecting immune response and specific immune resistance mechanisms are multifaceted.

However, this study still has some limitations. Neural network algorithms have the disadvantages of black boxes, which are complex to explain with time-consuming training. Biomarkers such as tumor mutation burden (TMB) were not included. Additionally, this model requires more multi-center prospective data to validate. Further study is needed to validate the advantages of deep learning networks in immunotherapy predictive models.

## Conclusion

In conclusion, this study found that the neural network model based on clinical information can accurately predict the efficacy benefits of ICI therapies for lung squamous carcinoma patients, especially DCR and ORR. This novel predictive model may provide reliable individual response information and treatment recommendations. In our retrospective study, we found that neoadjuvant and adjuvant immunotherapy may bring greater efficacy benefits to patients.

## Data availability statement

The raw data supporting the conclusions of this article will be made available by the authors, without undue reservation.

## Ethics statement

This research has been approved by the Ethics Committee of Beijing Chest Hospital affiliated with Capital Medical University. Given that this was a retrospective analysis, individual consent was waived.

## Author contributions

JW, SL, WL and TM came up with the idea for the study. SL, TM, SF, XG, NQ, YW, XZ and JW collected the data and conducted the follow-up. WL carried out the statistical analysis. SL, TM and WL wrote the manuscript. JW, ZL and YP edited and reviewed the manuscript. All authors discussed the results and commented on the manuscript. All the authors contributed to the article and approved the submitted version.

## Conflict of interest

The authors declare that the research was conducted in the absence of any commercial or financial relationships that could be construed as a potential conflict of interest.

## Publisher’s note

All claims expressed in this article are solely those of the authors and do not necessarily represent those of their affiliated organizations, or those of the publisher, the editors and the reviewers. Any product that may be evaluated in this article, or claim that may be made by its manufacturer, is not guaranteed or endorsed by the publisher.

## Supplementary material

Supplementary Figure 1 The distribution of missing values of clinical features in this study. PD-L1, programmed cell death ligand 1. BOR, best of response. ORR, objective response rate. DCR, disease control rate. PFS, progression-free survival. OS, overall survival.

Supplementary Figure 2 The training curves of **(A)** DCR model, **(B)** ORR model, **(C)** PFS model and **(D)** OS model. DCR, disease control rate. ORR, objective response rate. PFS, progression-free survival. OS, overall survival.

Supplementary Figure 3 The Kaplan-Meier curve compared the overall survival of patients with different gene mutations. Uncommon gene mutation.

Addition File 1 The original codes of DCR model, ORR model and PFS model. DCR, disease control rate. ORR, objective response rate. PFS, progression-free survival.

Addition File 2 The original codes of OS model. OS, overall survival.

Addition File 3 The weights and hyper-parameters of DCR model, ORR model, PFS model and OS model. DCR, disease control rate. ORR, objective response rate. PFS, progression-free survival. OS, overall survival.

Addition File 4 The clinical data used by this study.

## Abbreviations

AJCC, American Joint Committee on Cancer; AI, artificial intelligence; ALK, anaplastic lymphoma kinase; AUC, area under the receiver operating characteristic curve; BOR, best overall response; CI, confidence interval; CR, complete response; DCR, disease control rate; DNN, dense neural network; EGFR, epidermal growth factor receptor; IHC, immunohistochemistry; MPR, major pathological response; NSCLC, non-small cell lung cancer; ORR, objective response rate; OS, overall survival; PD, progressive disease; PD-1, programmed cell death 1; PD-L1, programmed cell death ligand 1; PFS, progression-free survival; pCR, pathological complete response; PR, partial response; SD, stable disease, TPS, tumor proportion score.
