# Supplementary material for: Assessing the efficacy of immunotherapy in lung squamous carcinoma using artificial intelligence neural network
Source: Front Immunol. 2022 Nov 28;13:1024707. doi: 10.3389/fimmu.2022.1024707 (PMC9742243; doi:10.3389/fimmu.2022.1024707)
Supplement: Supplementary file 1 [file DataSheet_1.pdf]

DCR model

OrderedDict([('net.0.linear.weight', tensor([[-6.6847e-01, 1.6315e-01, 2.2590e-02, 8.8139e-01, 2.9359e-01,
3.2289e-01, -1.0754e+00, 1.6577e+00, 3.3984e-01, 9.0125e-01,
-3.5496e+00, 1.8578e-01, -1.5200e+00, 4.9048e-02, 3.0729e+00,
2.3959e+00, 4.0145e+00, -1.3840e+00, -1.3766e+00, 2.8588e+00,
-1.1868e-01, 5.6826e-01, 9.9726e-01, -5.7846e-01, -1.6100e-02,
-3.4798e-01, -1.9679e-01, -3.0737e-01, -1.2283e+00, 3.1941e-01,
1.8800e-01, 8.9182e-01, 7.4462e-01, 2.6446e+00, -8.9286e-01,
7.1064e-02, 5.2518e-01, 1.2531e+00, -7.8242e-01, -1.4719e-01,
3.1190e-02, 8.8265e-01, -1.9395e-01, 9.9647e-01, -5.6098e-01,
-1.3310e+00, 1.3508e+00, -1.0309e+00, 2.4282e+00, -5.5627e-01,
2.3338e+00, -1.1218e+00, 7.1112e-01, 7.8196e-02, 8.7542e-01,
-6.6051e-01],
[-5.6096e-01, 3.6876e-01, 8.4709e-01, -4.0769e-01, 7.5036e-01,
-1.1071e+00, 9.9210e-01, 3.0979e-01, -2.1267e+00, -1.7280e+00,
2.3451e+00, -1.4236e+00, 7.0804e-01, -7.2444e-01, -2.5812e+00,
1.7818e-01, -3.7845e+00, -1.8746e-01, 7.0751e-01, -4.3755e+00,
1.6739e-01, 8.6648e-02, 4.3104e-01, 6.3838e-01, -5.7032e-01,
-5.0483e+00, 2.5815e-01, -2.8798e-01, 9.3239e-01, -1.5553e-01,
-1.7122e+00, 1.0115e+00, -1.1291e-01, -3.3711e+00, -9.7836e-01,
1.4820e+00, -3.7411e+00, -1.5273e+00, 1.9995e+00, 3.6384e-02,
-6.5873e-01, 1.6547e-01, -1.3108e+00, 8.0567e-01, -3.0004e+00,
-3.5850e-01, -2.0048e-01, 8.1362e-01, -2.6175e+00, 4.1063e-01,
-1.4376e+00, 2.6599e+00, 9.8384e-01, -1.9856e+00, -1.6982e+00,
1.5923e+00],
[5.0991e-01, 4.8621e-01, 6.7186e-02, 1.9225e-02, -1.9669e-01,
-4.2812e-01, -2.0689e-02, -1.5741e-01, -3.2073e-01, -9.0004e-02,
2.3353e-01, 8.0581e-02, -4.3114e-01, 8.0508e-02, 3.0192e-02,
-1.5049e-01, 1.9338e-01, 7.6299e-02, -1.9351e-01, -8.0069e-02,
6.4317e-01, -4.3777e-01, -3.4047e-01, 4.0961e-01, -1.6914e-01,

```
  2.1441e-01, -3.4903e-01,  5.5579e-02, -1.2385e-01,  1.2748e-01,
  9.0231e-02, -3.8841e-01, -2.8740e-01,  4.7847e-02, -1.7852e-01,
 -6.9305e-01, -1.9861e-01, -1.4187e-01, -1.0901e-01, -2.6561e-01,
  6.8131e-01, -3.3853e-01, -2.0760e-01, -1.9280e-01,  1.1736e-02,
 -5.2948e-01,  6.6044e-02, -4.2869e-01, -7.5813e-02,  1.0868e-01,
 -5.3614e-01,  7.0923e-02, -2.0904e-01,  5.8171e-01, -3.8737e-01,
  9.6328e-02],
[-7.9833e-02, -2.0957e-01, -3.3231e-01, -1.8623e-01, -4.0655e-01,
 -5.5717e-01,  9.5221e-03, -4.8041e-01, -1.7946e-01,  4.2762e-01,
  1.9468e-02, -6.4724e-02, -4.4646e-01,  8.1733e-02, -6.8073e-02,
  1.5509e-01,  2.6588e-01, -3.1228e-01,  6.6129e-01, -1.6099e-01,
 -4.3456e-01, -4.0000e-02, -4.1442e-01, -1.2703e-01,  1.4027e-01,
 -4.6875e-01,  1.2771e-01, -2.3842e-01,  4.9530e-01,  3.7329e-01,
  6.3422e-01,  9.2222e-02,  1.8490e-01, -1.1572e-01, -3.1548e-01,
 -4.5900e-01, -3.3254e-01,  1.2563e-01, -7.3057e-02, -4.4327e-01,
 -3.0956e-01, -4.9393e-01,  3.9449e-01, -3.9499e-01, -2.7486e-01,
 -2.0626e-01, -5.5288e-01, -4.0922e-01,  3.3510e-01, -2.2633e-01,
 -6.0690e-01,  4.6672e-03, -5.9403e-01, -3.3198e-01, -4.9565e-01,
 -1.5093e-01],
[ 1.1183e-01,  6.0887e-01, -4.7441e-01, -5.5066e-01, -8.8094e-02,
 -3.7592e-01,  6.3366e-01, -3.8874e-01, -3.6771e-01, -1.2569e-01,
 -3.5057e-01, -9.7694e-02, -2.1990e-01,  7.9781e-02,  2.8759e-01,
  2.6885e-02, -6.8559e-02, -2.5029e-02, -3.7486e-01,  4.6006e-01,
 -7.9015e-01, -3.8111e-01, -3.2490e-01, -1.4607e-01, -3.9126e-01,
 -2.8265e-01, -3.5155e-01,  5.1400e-02, -2.6242e-03, -1.4416e-02,
  9.7528e-03, -1.0910e-01, -4.8597e-01, -3.6248e-01, -5.1025e-01,
 -2.4654e-01, -3.1945e-01, -5.7857e-01, -1.9630e-01, -6.6314e-01,
 -3.9814e-01, -3.3077e-01, -4.8513e-01, -2.8096e-01,  2.9807e-01,
 -1.9026e-01, -5.1477e-01, -4.4727e-01, -5.1736e-01, -3.8552e-01,
 -4.6042e-01,  9.1915e-02, -4.2218e-01,  3.7353e-02, -1.9708e-01,
```

-5.2607e-01],
[8.2631e-01, 5.2880e-01, 3.6106e-01, 5.4647e-01, 2.4442e-02,
6.1179e-01, -6.8123e-02, -9.6858e-01, 1.3542e+00, 2.7130e+00,
-8.7593e-01, -3.7134e-01, -2.2413e+00, 1.0916e+00, 7.2470e-01,
8.6674e-01, 5.2398e-01, 1.1354e+00, 8.6068e-01, 1.7214e+00,
1.8366e-01, -8.5860e-01, 3.1841e-01, -3.7149e+00, -4.9881e-02,
9.0310e-01, 1.9333e+00, -1.2875e+00, 5.9273e-01, 2.9457e-01,
-2.9695e-01, -5.2967e-01, 3.0173e-01, 3.4306e+00, -3.2637e-01,
7.0141e-01, -2.6332e-01, 1.0631e-01, 4.9353e-01, 5.9945e-01,
4.3084e-01, 2.3029e-01, 9.3873e-03, 3.0098e-01, 5.5094e-01,
1.7207e-03, 8.2650e-01, -1.3059e+00, 3.1516e+00, -1.3526e+00,
1.3963e+00, 3.7812e-01, -7.5184e-01, 1.4439e+00, 1.6577e+00,
-1.6730e+00],
[6.4770e-01, 2.6462e-02, 8.1451e-01, -3.2448e-01, 1.1525e+00,
4.9256e-01, -1.8333e+00, 1.3824e+00, 1.7712e+00, 1.1938e+00,
-3.5373e-01, 1.2355e+00, -9.3127e-01, -2.7410e-01, 2.0023e+00,
-8.0410e-01, 1.7046e+00, 1.1184e+00, -1.1436e+00, 3.8994e+00,
-5.1357e-01, -6.6373e-01, -1.5279e+00, -1.5272e+00, 2.1399e+00,
8.5951e-01, 4.0121e-01, -1.2802e+00, -1.2393e+00, 2.1920e-01,
2.0439e+00, 1.2980e-01, 3.5626e-02, -1.0698e+00, 2.0599e+00,
6.8983e-01, -4.7085e-01, 1.4904e+00, -8.2862e-01, -8.5613e-01,
6.1654e-01, 7.5490e-01, 4.8717e-01, 1.1758e+00, -3.1278e-01,
-3.1545e-01, 6.4604e-01, -1.6591e+00, 2.8089e+00, -8.0607e-01,
5.5739e-01, -4.1395e-01, 5.5917e-02, 1.0889e+00, 3.7133e+00,
-2.5193e+00],
[1.8665e-01, -1.7738e+00, -1.2567e+00, 2.1062e+00, 1.6310e+00,
-6.8070e-01, 1.0884e-01, 1.0503e+00, -6.6988e-01, 1.2239e+00,
-3.2842e+00, -8.8026e-02, 7.2711e-01, -3.6174e+00, 7.7145e-01,
1.4846e-01, 3.3768e+00, 8.9720e-01, -2.1887e+00, 2.1820e+00,
2.3705e-01, 1.1648e+00, 6.6400e-01, 3.4458e+00, 1.0467e+00,

-2.7305e+00, -9.9674e-01, 1.0024e+00, -2.2636e+00, -5.4504e-01,
-1.1009e+00, 1.1204e+00, 9.4823e-01, 1.8584e+00, 3.9992e-02,
-6.4295e-01, 6.0227e-01, 1.5795e+00, -7.9816e-01, -4.5410e-01,
9.6222e-01, 1.4685e+00, -6.3219e-01, 1.0427e+00, -1.0834e+00,
-1.9461e+00, 2.2338e+00, -8.8805e-01, 2.4357e+00, -5.5019e-01,
1.4805e+00, -2.5122e+00, 7.5730e-01, -8.2022e-01, 1.6665e+00,
-1.4999e+00],
[1.1566e+00, 1.8095e+00, -5.4216e-01, 1.6194e-01, -2.2553e+00,
8.3434e-01, -4.6855e-02, 3.7890e-02, -3.8954e-01, 2.0877e+00,
-5.7222e-01, 1.6119e+00, 3.2887e+00, 6.0797e+00, 2.5803e+00,
-1.5427e+00, -4.6538e-01, -6.3896e-01, 3.1698e-01, 2.1900e+00,
-2.0292e+00, 6.9663e-01, 3.6472e-02, 3.6265e+00, -4.0861e-01,
1.0941e+00, -9.6463e-01, -7.4603e-01, 3.3465e-01, 1.0968e+00,
6.1369e-01, -8.3238e-01, -1.3963e+00, 4.3012e+00, -1.4074e-01,
3.5485e-01, -1.5076e+00, 7.2930e-01, -2.4816e+00, -1.6924e-01,
-1.8183e-01, 1.2148e-01, 2.6534e-01, -1.3260e-01, 5.6359e-01,
7.2431e-01, 8.8761e-02, 1.9797e-01, 2.7456e+00, 1.0931e+00,
-1.1527e+00, -9.9505e-01, -9.4456e-01, 1.6075e+00, -6.9440e-01,
2.8161e-01],
[6.6018e-03, 3.0417e-01, 5.8678e-01, -9.5411e-01, 4.6592e-01,
-9.5978e-01, 7.2336e-02, 6.3485e-01, -9.9468e-01, -6.6145e-01,
1.4562e+00, -4.3169e-01, 1.2522e+00, -2.0258e+00, -1.5895e+00,
-1.1211e+00, -7.1433e-01, -6.3131e-01, -8.2648e-01, -1.7603e+00,
3.0067e-01, 1.2492e+00, 1.5176e-01, -7.3414e-01, -2.1997e-01,
-1.2998e+00, 1.1775e-01, -4.2587e-01, -1.1791e+00, 2.9323e-01,
2.4020e-01, -1.6329e-01, 6.8456e-01, -2.0652e+00, -7.0063e-01,
-2.9524e-02, -7.4748e-01, -1.2055e+00, 6.0485e-01, -7.8489e-01,
3.2127e-01, 6.4370e-02, -6.4785e-01, -5.3042e-01, 5.5439e-01,
-7.0297e-01, -9.4622e-02, 3.6833e-01, -1.9566e+00, 1.0514e-01,
-1.5389e-02, -7.9816e-01, -4.1971e-01, -5.8415e-01, -1.0760e+00,

6.3584e-01],
[4.1950e-01,   1.6314e-01,   1.1161e+00, -2.0016e+00, -6.4006e-01,
-3.9000e-01, -2.4639e-01, -2.9540e-01, -7.4123e-01, -2.8080e+00,
1.4289e+00, -8.4531e-02,   1.2120e+00, -4.1293e-01, -2.7774e+00,
-4.8252e-01, -1.7316e+00, -1.0203e+00, -1.9523e+00, -1.0677e+00,
7.5603e-01,   1.0079e+00, -5.1057e-01, -3.1940e+00, -9.1613e-01,
-9.4245e-02,   1.8484e+00, -1.1644e+00,   1.2948e+00,   6.3078e-01,
-2.4463e-01, -4.5302e-01, -4.1980e-02, -2.2460e+00,   7.0723e-02,
-9.0695e-01, -1.3080e+00, -2.4742e+00,   1.4318e+00, -8.5752e-01,
2.1315e-01, -2.2805e+00,   1.2193e+00, -1.4056e+00,   9.4385e-02,
2.2001e-01, -8.5207e-01,   5.9503e-01, -2.7448e+00, -3.1580e-02,
-6.2770e-01, -3.4379e-01, -1.0383e-02, -1.1752e+00, -1.9193e+00,
6.4747e-01],
[-3.7272e-01, -2.1156e-01,   2.0845e-01, -1.0950e+00, -3.0647e-01,
-1.1069e-01, -5.0415e-02, -8.9541e-01, -2.6291e-01, -2.7592e-01,
-4.2880e-01, -7.4405e-01, -6.2138e-01,   5.5158e-01, -1.9808e-01,
5.1800e-01, -1.4082e-01, -7.5122e-01, -6.6111e-01, -1.5708e+00,
1.1671e-02,   3.5608e-01, -4.2303e-01,   9.5508e-02, -9.6643e-01,
1.2031e-01,   1.5195e-01, -5.8938e-01,   5.7040e-02, -5.4456e-01,
-4.2310e-02, -4.9449e-01, -1.5854e-01,   1.8663e-01,   2.9143e-01,
-4.9234e-01, -1.1596e-01,   1.2389e-01, -2.9329e-01, -6.8908e-01,
1.3991e-01, -8.0161e-01,   9.4674e-01, -7.2618e-01,   8.1129e-01,
1.5792e-01, -7.0198e-01, -5.0017e-01, -1.0559e+00, -3.6427e-01,
3.6573e-01, -6.7572e-01, -8.5217e-01,   2.1129e-01,   3.0063e-02,
-2.1551e-01],
[-1.3239e-01,   3.4169e-01,   4.8977e-01, -1.1509e+00, -1.8453e-01,
-8.3814e-01, -2.6238e-01, -5.9615e-01,   1.6659e-01, -6.9301e-01,
5.6051e-01, -7.7638e-01,   6.5366e-01, -4.6134e-01, -7.8268e-01,
-4.5378e-01, -9.0233e-01, -2.2611e-01, -1.0496e+00, -1.1411e+00,
9.1103e-01, -4.3722e-01, -2.1006e-01, -3.1600e-01, -6.2262e-01,

2.0678e-01,   8.1871e-01, -3.2742e-01, -2.7174e-01,   7.6621e-02,
1.8304e-01,   7.4849e-01, -9.4609e-01, -1.0067e+00,   1.7357e-02,
-6.0070e-01, -1.9640e-01, -9.0411e-01,   6.1262e-01, -8.6257e-01,
2.5562e-01, -8.9880e-01, -3.0766e-01, -2.0251e+00,   8.0580e-01,
-5.5082e-01, -5.5044e-01, -1.7871e-01, -1.2633e+00, -1.0288e+00,
4.1870e-02, -5.0339e-01, -7.3356e-01, -9.0427e-02, -2.3795e-01,
-1.9624e-01],
[-2.4158e-01, -3.9264e-01, -3.7486e-01, -3.7790e-02, -3.7524e-02,
-3.2978e-01, -2.6098e-01, -1.8477e-01, -6.6248e-01, -1.3521e-01,
-1.8791e-02,   2.6014e-02,   6.6934e-01, -1.3273e-01, -2.1541e-01,
-1.8420e-01, -2.0247e-01,   1.1264e-01,   7.4921e-02,   5.2631e-02,
-1.9366e-01,   3.7982e-01, -5.0403e-01, -1.1083e-01, -4.2510e-02,
-6.5980e-02, -1.3687e-01,   1.3260e-01,   3.0784e-01, -1.2627e-01,
-3.1922e-01, -1.5065e-01,   4.6685e-01, -1.2012e-01, -1.9476e-01,
-3.1455e-01,   5.0397e-01, -4.0306e-01, -6.5884e-02, -5.1218e-01,
-3.6105e-01, -5.8291e-01, -2.0156e-01, -4.8293e-01, -2.1654e-01,
-4.8769e-01,   2.1063e-01, -2.9039e-01, -3.9449e-01, -1.7909e-01,
-4.6341e-01,   4.2978e-02, -7.1376e-01, -4.6044e-01, -1.8232e-01,
-4.6301e-01],
[7.0791e-01, -9.5020e-01, -2.8204e-02,   4.5051e-01,   7.1137e-01,
2.8899e-01, -1.0711e-01, -2.1770e-01,   5.8466e-01,   9.0446e-01,
-1.7717e+00,   2.9859e-01, -2.3512e+00, -8.0345e-02,   2.9746e+00,
1.1670e+00,   2.7940e+00, -9.7982e-02, -1.8663e-01,   2.2406e+00,
8.3370e-01, -9.2992e-01,   8.6971e-01, -1.8108e+00, -8.6882e-01,
-7.9724e-01,   1.5931e+00,   7.2910e-01, -2.8615e-02,   6.7158e-01,
-5.1173e-01, -5.3079e-01,   6.6557e-02,   3.8062e+00, -2.7619e-01,
-4.9726e-01,   4.9772e-01,   2.1798e-01,   3.9834e-01,   7.0491e-01,
-6.6657e-01,   1.2755e+00, -1.0774e+00, -7.5219e-01,   1.0265e+00,
1.6737e-01,   3.6136e-01, -1.2040e+00,   2.9966e+00, -1.3431e+00,
2.6453e+00, -2.1250e+00, -9.1319e-01,   1.6879e+00,   1.2807e+00,

-1.5680e+00],

[-4.0977e-02, 9.5429e-01, -3.6772e-01, 6.7392e-01, -1.7318e-01,

6.8114e-01, 7.7035e-01, 3.4198e-01, -6.7430e-01, 1.1049e+00,

-1.8910e+00, 4.4209e-01, -8.6098e-01, 3.4296e-01, 2.4089e+00,

3.2432e-01, -1.5446e+00, 2.2342e-01, -5.1475e-01, 1.4310e+00,

-4.8507e-01, 1.4629e+00, -5.8780e-01, -8.5558e-01, 2.1291e-02,

1.2624e+00, 6.8968e-01, 2.5747e+00, -2.0900e-01, 1.2033e+00,

1.1593e+00, -9.2817e-01, 4.5791e-01, 2.1086e+00, 8.3029e-01,

7.4714e-01, -1.9299e+00, 9.6293e-01, -1.1229e+00, -3.2716e-01,

3.3985e-01, 1.2475e-01, 1.2908e-01, 3.6041e-03, 6.4378e-01,

3.0068e-01, 6.4157e-01, 1.4865e-01, 1.0240e+00, -8.0716e-01,

4.3344e-01, 1.6527e-02, 3.0366e-01, -3.9539e-01, 2.4440e-01,

4.3810e-01]])), ('net.0.linear.bias', tensor([0.6067, -0.3372, -0.3286, -0.4313, -0.
4690, 0.9086, 0.2991, 0.3933,

-0.0287, -0.8279, -1.5083, -0.6932, -0.9810, -0.5335, 0.4018, 0.3747])), ('net.0.b
atch_norm.weight', tensor([2.2393, 2.0995, 2.9957, 0.4897, 1.0683, 2.5563, 0.4375, 2.3580,
2.1195,

1.8556, 1.7463, 0.5231, 1.0483, 1.3172, 2.2046, 0.8160])), ('net.0.batch_norm.bias',
tensor([1.8595, -0.6212, -0.0582, -0.1011, 0.2093, 0.7495, 0.1808, 0.6385,

-0.0487, 0.5181, -0.5310, 0.6227, 0.4218, 0.1033, -0.1104, 0.1880])), ('net.0.
batch_norm.running_mean', tensor([6.3903e+00, 3.2745e-01, 1.3262e-02, 7.3936e-33, 8.3776e
-33, 6.0203e+00,

6.7178e+00, 6.9745e+00, 1.7916e+00, 2.0896e-03, 1.6317e-08, 6.0065e-22,

4.3031e-24, 6.4311e-33, 4.3655e+00, 4.6209e+00])), ('net.0.batch_norm.running_var
', tensor([1.0289e+01, 1.2571e+00, 1.8441e-02, 4.1715e-31, 4.1794e-31, 9.7681e+00,

1.6293e+01, 1.3175e+01, 6.8791e+00, 1.4626e-03, 4.8174e-08, 8.5384e-22,

1.0958e-23, 4.1655e-31, 1.0819e+01, 7.8427e+00])), ('net.0.batch_norm.num_batche
s_tracked', tensor(664)), ('net.1.linear.weight', tensor([[0.4579, -1.4165, -1.4501, -0.3649, -0.
4248, 1.5363, 0.7878, 0.7995,

-0.0120, -1.8409, -0.1379, 0.3811, -0.5095, -0.2362, 2.0266, 0.2086],

[-2.3833, 0.2125, 0.7484, -0.7715, 1.6078, 0.2858, -1.0215, -2.8539,
-0.4654, 0.7834, 1.8047, 0.4795, 1.0300, -0.6725, -0.1384, 0.0134],
[-0.1625, 1.1679, 1.4487, -0.2971, 0.4266, -1.2440, -0.5101, 0.3367,
-1.2703, 1.6530, 0.3525, 0.4444, 0.5090, -0.0394, -0.8003, -0.6648],
[0.2196, -2.0710, -1.4982, 0.4949, 0.6385, 1.8904, 0.8078, -0.1486,
1.8790, -0.3903, -0.9868, 0.7742, -0.2305, -1.1451, 0.7776, 1.7054],
[1.0656, -1.2917, -0.0532, 0.4697, -0.4467, 1.5061, -0.9649, 0.2624,
-2.1820, -0.2610, -0.5534, -0.8466, 1.2582, 0.2026, 1.5242, 0.1940],
[-2.5892, 0.8686, 1.6723, -0.1712, -0.6422, 0.0608, 0.5105, -1.3784,
-2.0262, 0.1654, 1.9450, 0.6231, 0.3627, 0.0669, -0.3897, -0.7132],
[1.4140, -1.5814, -0.3418, -1.4527, -0.0091, 2.4140, 0.8602, 0.3248,
-2.3651, 0.1119, -0.7948, -0.4864, 0.6694, -0.5873, 1.7294, -0.8055],
[1.0583, -1.7913, -0.6543, -1.0313, 0.8706, 2.1564, -1.4011, 0.3718,
0.7565, -0.3990, -0.2359, 0.3886, -0.1856, 0.3164, 1.8868, 0.5296]])), ('net.
1.linear.bias', tensor([1.0836, 0.8301, 1.0257, 0.3585, 0.0450, -0.1739, 1.3378, 0.996
8])), ('net.1.batch_norm.weight', tensor([1.0617, 0.4920, 0.6560, 1.3208, 0.9183, 0.8548, 0.80
53, 1.1054])), ('net.1.batch_norm.bias', tensor([1.0287, -0.9857, -0.8290, 0.7042, 1.1992,
-0.8687, 0.8706, 1.2267])), ('net.1.batch_norm.running_mean', tensor([6.3206, 2.2244,
2.9654, 7.0079, 7.1482, 1.6189, 11.4988, 8.2864])), ('net.1.batch_norm.running_var', ten
sor([52.2551, 19.0791, 26.9935, 61.0039, 56.3902, 16.2272, 119.3934,
83.7237])), ('net.1.batch_norm.num_batches_tracked', tensor(664)), ('net.2.weight', t
ensor([[0.5188, -0.3574, -0.2208, 0.4526, 0.5613, -0.3920, 0.3480, 0.6030]]))])

ORR model

```
OrderedDict([('net.0.linear.weight', tensor([[ 2.7366e-01,  2.6185e-01,  1.5165e-01,  2.1128e-01,  4.9188e-01,
          6.5851e-02, -2.3341e-01,  1.0280e+00,  3.1072e-04, -1.2871e+00,
         -1.7786e+00,  1.3774e+00,  1.3805e-01,  1.1147e+00,  7.8142e-01,
         -1.4272e+00,  5.1304e-01,  3.3618e-01,  1.0432e-01,  1.9356e-01,
         -2.3355e-01,  2.9739e-01,  3.1021e-01,  1.3362e+00, -8.4247e-02,
          8.1407e-01,  2.6318e-01,  1.2299e+00,  2.1948e-01,  8.6952e-02,
         -2.2445e-01, -6.9629e-02, -5.3143e-02,  6.7192e-01,  4.7260e-01,
         -5.0998e-01,  1.4448e+00,  1.1611e+00,  2.1003e-02,  6.6922e-01,
         -5.9497e-02,  3.7837e-01,  4.1003e-01,  1.9835e-01,  1.6729e-01,
          2.9907e-01,  6.4873e-01,  7.3712e-01, -3.4091e-01,  6.9993e-01,
         -4.5444e-01,  1.0945e+00,  1.1231e+00, -7.4562e-01, -2.1665e-01,
          6.8740e-01],
        [ 5.2025e-01, -2.0537e-01, -2.5971e-01,  1.0699e+00,  2.3151e-02,
          2.8860e-01,  5.0614e-02, -4.7949e-01,  4.8958e-01,  7.9452e-01,
         -7.4018e-01, -2.0639e+00, -2.6907e-01,  7.9626e-01,  7.3633e-01,
         -5.5392e-03, -9.3849e-01,  1.5052e-02, -4.5981e-01,  8.3200e-01,
         -3.7729e-01,  7.8022e-01,  3.9017e-01, -3.5560e-01, -2.8078e-01,
          2.1482e-01,  4.1247e-02, -1.6751e+00,  6.9172e-01,  1.5066e+00,
         -3.1864e-02,  2.4471e-01, -1.1779e+00,  9.8175e-01, -6.6226e-02,
          1.3018e+00, -5.7867e-01,  1.2660e+00, -7.8109e-01, -5.7139e-01,
          8.8452e-01,  7.3105e-01, -5.9230e-01,  1.1665e+00, -5.7768e-01,
          1.0189e+00, -5.0762e-01, -1.0776e+00,  1.5636e+00,  1.2157e+00,
         -7.7047e-01,  6.0772e-01, -3.3762e-02,  4.0437e-01,  1.3593e+00,
         -1.1002e+00],
        [-4.4144e-01,  3.0647e-01, -3.2457e-01, -6.3546e-01,  3.8609e-02,
         -4.5588e-01,  4.7950e-01, -3.8260e-01,  3.0759e-01, -1.0516e-01,
          5.3751e-01,  6.2562e-02,  2.9294e-01,  7.0168e-01, -4.5275e-02,
         -1.4444e-01,  1.1631e-01, -7.1190e-02,  1.7070e-01, -2.4934e-01,
         -2.3061e-01, -3.0914e-01, -1.4272e-01,  2.4469e-01, -1.3586e-01,
```

-1.6378e-01, -6.2705e-01, -4.3905e-03, 1.2213e-01, 6.7804e-02,
1.6108e-02, -2.5768e-01, -1.8368e-01, 5.9690e-02, -1.9918e-01,
-6.8137e-02, -1.4754e-01, -1.4135e-01, 2.3881e-02, 3.6861e-01,
-4.7494e-01, -3.0438e-01, -8.2669e-02, -3.2353e-01, -3.4924e-01,
2.6794e-01, -6.6379e-02, -3.8719e-01, -2.9584e-02, 1.8159e-01,
-2.6495e-01, -1.7369e-01, -2.5755e-01, -3.0954e-01, 1.3976e-01,
8.2908e-02],
[-4.4562e-01, -5.8143e-01, 3.4014e-01, 3.8962e-01, 9.4540e-02,
-1.8874e-02, 5.9632e-02, 1.4347e-01, 2.4374e-01, 5.9665e-02,
4.0937e-01, -4.5839e-01, 1.7955e-01, 1.6294e-01, 1.3328e-01,
-6.1887e-02, 5.8168e-01, 2.8990e-01, 5.9266e-01, -4.8304e-01,
-9.1621e-02, 6.1492e-02, 8.4167e-02, 7.9745e-01, 3.9994e-01,
9.6924e-02, -7.1364e-01, -6.2600e-02, 3.5517e-01, -1.5770e-01,
1.6102e-01, -4.8183e-01, 5.6485e-01, -1.6810e-01, 2.1897e-01,
-3.3052e-02, 3.0210e-01, -1.6878e-01, 4.1870e-01, 2.3684e-01,
-5.8842e-01, 3.6802e-01, 1.7630e-01, 3.9374e-01, -1.1498e-01,
3.5720e-01, -3.1384e-01, 5.8935e-01, -3.0583e-01, -7.5108e-02,
3.7786e-01, 1.3964e-01, -2.6787e-02, -1.7632e-01, 2.0307e-01,
-6.5758e-01],
[-9.5810e-03, -1.5061e+00, -9.0637e-01, 5.3391e-01, -1.1294e+00,
4.8565e-01, 7.9343e-01, -8.6088e-01, 3.1523e-01, 3.4716e-01,
1.0649e+00, -5.5314e-01, -1.8580e-01, -7.5265e-01, -7.9374e-03,
6.4404e-01, -1.0657e+00, -1.6186e-01, -6.4829e-01, -1.0303e+00,
-3.0620e-01, 1.6748e+00, -8.3254e-02, -8.5037e-01, 9.9781e-01,
8.2676e-01, -1.1398e+00, -5.4940e-01, 2.1601e-02, 1.3371e+00,
6.4206e-01, -6.8214e-01, -6.2425e-02, 9.4498e-01, 3.8263e-01,
7.2435e-01, -8.1282e-01, 9.9637e-02, 6.0875e-02, -6.9833e-01,
6.8487e-01, 1.7295e-03, -8.5586e-01, 1.8051e-01, -2.3716e-01,
-2.3328e-02, 2.4670e-01, -6.2874e-01, 1.1534e+00, -3.6211e-01,
4.2001e-01, -1.0564e-01, -3.1905e-01, 3.2775e-01, 1.1402e-01,

-1.0660e+00],

[-1.5652e-01, -9.0409e-01, -3.5209e-01, 2.8191e-01, -6.4199e-02,

-4.8851e-01, -9.7254e-01, -2.5196e-01, 1.2261e+00, 1.0654e+00,

3.4202e-01, -7.2366e-01, -4.2964e-02, 4.1914e-01, 4.1010e-01,

-1.2539e-01, -5.1155e-01, 7.6065e-02, -5.2224e-01, -4.0692e-01,

5.2410e-01, 2.9767e-01, -2.8375e-01, 4.3576e-01, -2.7131e-01,

-4.6431e-01, 2.2860e-01, -4.4228e-01, 6.5566e-01, 8.7692e-01,

-4.0214e-01, -3.5106e-01, 2.8893e-01, 7.5959e-02, -1.8519e-01,

-3.8603e-01, 5.0717e-01, -2.4210e-01, 1.0637e-01, 2.0042e-01,

-5.4984e-02, -5.4536e-02, -7.8631e-01, -5.5512e-03, -2.4369e-01,

-3.7824e-01, 2.6370e-01, -2.4531e-01, 6.4419e-01, -2.6210e-01,

4.3672e-01, 1.2460e+00, -4.2949e-02, 1.1236e-01, 1.1352e+00,

-7.7910e-01],

[5.9855e-03, 2.6945e-01, -4.7907e-01, 8.3366e-02, 3.5836e-01,

-2.2961e-02, 5.8362e-01, 6.5865e-01, -8.9812e-01, -4.2066e-01,

-1.0281e+00, 1.8102e+00, -3.3150e-01, 4.8803e-01, 2.8548e-02,

-2.0620e-02, 3.9463e-01, 8.1271e-02, -3.3355e-01, 5.6828e-02,

-1.1566e-01, 4.3671e-01, 6.1904e-01, 9.1566e-03, -2.1592e-01,

-3.0167e-01, -2.1239e-01, 2.2621e+00, -6.4707e-01, -9.6883e-01,

9.6380e-02, 6.5485e-02, 2.0774e-01, 4.9879e-01, -4.0852e-01,

5.8110e-02, -3.6675e-01, 3.5325e-02, -9.0299e-02, 2.7004e-01,

-6.2824e-01, -2.6980e-01, 6.7096e-01, 3.8020e-01, -3.7232e-01,

-7.9611e-02, 3.6537e-01, 1.9643e-01, -7.7680e-02, 1.5143e-01,

2.2225e-02, 3.1236e-01, 6.3654e-01, -8.2245e-01, -3.2440e-01,

3.1598e-01],

[7.4867e-01, 8.0454e-01, -3.1314e-01, -1.6040e-01, -2.6550e-01,

-1.6268e-01, -5.9470e-01, 4.4116e-01, -4.6929e-01, -1.7755e-01,

2.1997e-02, 1.3546e-01, -3.6982e-01, -6.1314e-04, -1.5903e-01,

5.0685e-01, -3.1354e-02, -3.4083e-02, -1.1511e-01, -3.6622e-01,

-6.6544e-02, 1.4287e-01, -1.0498e+00, 1.0738e-01, -1.9417e-01,

1.1611e-01, 1.5647e-01, -7.5862e-02, -3.5856e-02, -1.1387e-01,
-1.1961e-01, 1.7987e-01, -2.7770e-01, -8.6079e-01, 2.0467e-01,
-4.5003e-01, -8.9518e-01, -2.5690e-01, -6.6180e-01, -1.9275e-01,
-7.7464e-01, -8.6633e-01, -4.0985e-01, -9.7512e-01, 1.1224e-01,
-1.7467e-01, -7.8254e-01, -7.7073e-01, -2.9213e-01, -2.0327e-01,
-1.0125e+00, -1.0573e-01, -4.8917e-01, 5.6433e-01, -1.3737e-01,
9.5064e-02],
[1.0241e+00, 8.4214e-02, 3.4608e-01, 3.7379e-01, 5.1730e-01,
1.9070e-01, -3.5921e-01, -8.6615e-02, 7.1815e-01, 3.7696e-01,
-6.6552e-01, 1.0152e-01, 6.3787e-01, -4.3727e-01, 3.3848e-01,
-5.3752e-01, -7.0754e-01, 2.2036e-01, -3.2324e-01, -4.3181e-01,
-2.2484e-01, 4.3771e-01, 1.9798e-01, -6.6387e-01, -8.0543e-02,
2.7722e-01, -1.2418e-01, 4.4405e-01, -2.6519e-01, 2.4351e-01,
-9.5460e-01, 7.6033e-02, -6.7397e-01, 6.6419e-01, -1.3324e-01,
3.5926e-02, 4.5529e-01, 6.8768e-01, -5.1129e-01, -2.9692e-01,
5.7343e-01, 1.2019e+00, -9.2509e-01, 4.6211e-01, 3.4326e-02,
3.9622e-01, 9.6640e-04, -5.6924e-01, 7.9760e-01, 9.5376e-01,
-5.3092e-01, 2.7710e-01, -1.2263e-01, 6.8746e-01, -1.5127e-01,
-1.6845e-01],
[8.4581e-02, -1.2619e-01, 2.3432e-01, 3.6867e-02, 6.6175e-02,
-1.9659e-01, -6.3813e-02, 1.4284e-01, 6.0538e-02, 7.3311e-01,
-4.9257e-02, 4.5597e-01, 3.1459e-01, -2.0984e-01, -2.2443e-01,
5.5487e-01, 4.7832e-02, 1.3874e-01, 3.6258e-01, -4.2141e-01,
-2.5190e-01, 8.0861e-02, -8.0458e-01, -5.0835e-01, 1.5342e-01,
1.1688e-01, 3.2756e-01, -1.2375e-01, 6.2548e-02, -9.1066e-03,
2.2620e-01, -5.2803e-01, -7.0376e-01, -4.7647e-01, 2.0735e-01,
-2.9948e-01, 2.8944e-01, -4.4279e-02, 2.3469e-01, -4.1471e-01,
4.2994e-02, -1.0443e+00, 1.4127e+00, -1.5418e-01, 7.6091e-01,
-7.1586e-01, 8.7487e-01, 7.4512e-02, 3.8884e-02, -1.9382e-01,
5.6023e-01, 5.0966e-01, -6.6127e-02, -2.1675e-01, -5.9924e-01,

```
  2.8795e-02],
[ 3.6239e-01,  5.4755e-02, -2.3727e-01, -2.5326e-02,  6.3404e-02,
  3.6019e-01, -3.2328e-01,  1.4727e+00,  2.8881e-01,  2.1321e-01,
  6.1235e-01, -3.8870e-01,  4.8984e-01,  1.1856e+00, -9.3119e-01,
 -5.8400e-01,  1.2300e-01, -1.4392e-01, -1.3965e+00, -2.6516e-01,
  8.0164e-01, -9.2108e-01,  1.1034e-02, -8.6450e-01, -3.3460e-01,
  1.8438e-02,  1.0032e+00, -4.1701e-02,  1.0149e-01,  1.5491e-01,
 -4.8008e-01, -2.6329e-01,  4.8503e-02,  2.5398e-01, -5.4950e-01,
  1.9236e-01, -2.9647e-01, -2.4620e-02, -3.3352e-01,  2.1177e-01,
 -3.2350e-01,  1.6643e-01,  1.0469e+00,  6.9447e-02, -1.3649e+00,
  1.9200e-02,  1.0683e-01,  2.0586e-01, -1.6561e-01,  1.4326e-01,
 -4.8707e-01, -1.4578e+00,  1.8918e-01, -1.1799e+00, -6.7050e-01,
  4.2211e-01],
[ 2.7539e-02,  1.8203e-01,  6.7297e-01,  1.7992e-01,  2.4853e-01,
 -4.2872e-01,  8.7574e-01, -7.1166e-01, -2.3339e-01, -4.0234e-01,
 -8.2209e-01, -1.4133e-01,  4.2813e-01, -4.7739e-01,  5.6091e-01,
  5.6568e-01, -1.0655e+00, -5.0910e-01,  4.5204e-01,  1.3157e-02,
 -2.6595e-01,  7.8296e-01,  2.1404e-01, -3.5503e-01, -2.4276e-01,
  8.6767e-02,  1.0248e+00,  1.5608e+00, -5.2271e-02, -7.3398e-02,
  1.5198e-01,  2.1474e-02, -1.3059e-01,  1.8295e+00, -2.1525e-01,
  3.1067e-01,  4.8736e-01,  9.4812e-01, -9.3869e-01, -2.6968e-01,
  1.1196e+00,  1.2078e+00, -1.2535e+00,  8.3202e-02,  3.3689e-01,
  4.6329e-01,  5.9073e-01, -2.1525e-01,  7.1560e-01, -8.1403e-01,
  1.1192e-01,  1.5372e+00, -4.9470e-01,  1.1695e+00,  1.3061e+00,
 -1.4905e+00],
[ 2.7296e-01,  1.6640e-01, -5.5192e-02, -5.8235e-01, -1.9319e-01,
 -2.8796e-01, -1.8204e-01,  1.2179e-01, -5.2938e-01,  2.9772e-01,
  1.1449e+00, -4.1625e-01,  2.1480e-01, -4.4075e-01,  8.8212e-02,
 -5.5267e-01,  2.9951e-01,  4.2412e-02,  2.7587e-01, -4.0721e-01,
 -3.1564e-02,  1.1777e-01, -5.5321e-01,  6.1876e-02,  2.3135e-01,
```

6.2492e-01, 9.1873e-02, 4.1175e-02, -2.5381e-01, -8.3005e-02,
5.1999e-01, 1.6562e-02, -2.8612e-01, 6.5787e-01, 1.5815e-01,
-3.3471e-01, 5.5981e-01, -2.8860e-01, 7.1707e-01, -4.9944e-01,
2.3799e-01, -3.4324e-01, -5.4768e-01, -4.3655e-01, -4.6333e-01,
-4.3761e-01, 3.1759e-01, -2.4125e-01, 5.0324e-01, 1.6776e-01,
-2.2189e-01, -5.0946e-01, -1.2303e-01, -3.7436e-01, -3.4801e-01,
6.5502e-02],
[3.8395e-02, 1.8772e-01, 4.2232e-01, -5.4043e-02, 3.8448e-01,
1.7417e-01, 5.0511e-01, 9.1391e-01, -6.2974e-01, -2.5321e-01,
-3.3889e-01, -1.2897e-01, -4.6685e-01, 1.3655e+00, 2.3295e-01,
-1.2450e+00, 6.8875e-01, 4.4101e-01, -4.4740e-01, 7.1462e-01,
-1.2988e-02, -2.5293e-01, 1.4597e-01, 2.7951e-01, 2.9566e-01,
1.3605e-01, -3.0632e-01, -2.9879e-01, 5.9763e-01, 1.6791e-01,
1.2643e+00, 1.1217e-01, 1.7709e-01, -5.5806e-01, -2.2718e-01,
1.5331e-01, -3.8747e-01, 2.2013e-01, -7.7905e-02, -1.8879e-01,
4.1331e-01, -8.9100e-01, 9.5393e-01, 3.9267e-01, -9.1789e-02,
-4.9038e-01, 5.9140e-01, -1.7950e-02, -4.3123e-02, -3.0687e-02,
-1.5337e-01, -5.4091e-01, 2.1601e-01, -3.4894e-01, -1.1251e+00,
1.0277e+00],
[4.7125e-01, -3.6120e-01, 1.2336e+00, -1.4653e-01, -5.2052e-02,
7.0533e-02, 1.0659e+00, -3.0313e-01, -5.3025e-01, -9.0684e-01,
9.1387e-01, 1.0768e+00, -3.1019e-01, 2.6760e-01, 4.6616e-01,
3.4134e-02, 8.2945e-01, -2.7187e-01, 3.6076e-01, -5.2412e-01,
-2.3931e-01, 4.6680e-01, -6.5604e-01, -1.9787e-01, 1.0939e+00,
6.1788e-01, -6.5379e-01, 5.0364e-02, 3.7240e-01, -9.0543e-01,
-2.8931e-01, -3.3921e-01, -4.4827e-01, -1.9486e+00, 6.6692e-01,
-1.8488e-01, 1.2651e+00, -3.1475e-01, 3.4627e-01, 4.6662e-02,
5.1130e-01, -9.4618e-02, 4.7490e-01, 1.0675e-01, 5.7525e-02,
-3.1955e-01, 5.0100e-01, 2.7128e-01, -4.0906e-01, 3.2784e-01,
2.6396e-01, -5.1029e-01, 1.1160e-01, -5.9322e-01, -4.9928e-01,

8.4601e-01],

[-8.1914e-01, -1.6857e-01, 5.1181e-01, -3.0141e-02, 2.7138e-02,

-1.2721e-01, -1.6679e-01, -5.0194e-01, 2.7100e-03, -9.5137e-02,

5.0184e-02, 1.2193e-01, -3.9581e-01, 4.4264e-01, 3.8188e-01,

-1.1471e-01, -1.8191e-01, -3.0015e-02, -2.9166e-02, 1.9015e-01,

-9.7725e-01, 1.3282e-01, 7.5512e-02, 2.8825e-01, -7.2479e-01,

3.0454e-02, -6.8415e-01, 5.5235e-02, -3.3853e-01, -4.2773e-02,

5.2712e-01, -5.7564e-01, -4.5352e-01, -2.0083e-01, -1.6287e-01,

-1.7239e-01, -3.1692e-01, -6.1758e-02, -4.3555e-01, -2.2714e-01,

-5.2778e-01, -6.4466e-02, -1.9092e-01, -3.5327e-01, 3.5883e-01,

-2.5762e-01, -7.5377e-01, -3.3966e-01, -1.6480e-01, -4.8015e-01,

-8.2026e-02, -2.6533e-01, -1.6435e-01, -4.1872e-01, -7.8666e-02,

-1.8193e-01]]])), ('net.0.linear.bias', tensor([0.6643, 0.7055, -0.3790, 0.3977, 0.0240, -0.1738, 0.0797, -0.6793,

0.3984, -0.0552, 0.0982, 0.6596, -0.6798, -0.0848, 0.2411, -0.1965])), ('net.0.batch_norm.weight', tensor([0.9583, 1.9279, 1.3003, 0.1149, 1.7787, 1.3648, 1.0790, 1.3647, 0.8859,

0.6433, 1.3343, 0.8252, 0.8753, 1.5677, 1.5434, 1.3326])), ('net.0.batch_norm.bias', tensor([-0.6034, 1.1017, -0.3077, -0.4588, 0.2807, 0.1543, 0.0510, 0.3019,

0.1417, -0.2202, 0.1778, -0.7907, -0.1060, -0.2135, -0.2151, -0.5961])), ('net.0.batch_norm.running_mean', tensor([4.6591e+00, 6.2502e+00, 3.7192e-10, 3.0417e+00, 2.0396e+00, 1.3453e+00,

9.6901e-01, 2.9623e-03, 2.6167e+00, 1.9522e-01, 6.1702e-01, 4.0697e+00,

9.8834e-04, 4.1680e-01, 4.5054e-01, 2.6544e-03])), ('net.0.batch_norm.running_var', tensor([3.6863e+00, 4.8309e+00, 2.5764e-07, 1.6471e+00, 2.7655e+00, 1.3543e+00,

9.8173e-01, 7.1218e-03, 2.3100e+00, 3.8279e-01, 7.3742e-01, 4.3292e+00,

2.6852e-04, 7.3230e-01, 1.0180e+00, 1.4116e-03])), ('net.0.batch_norm.num_batches_tracked', tensor(144)), ('net.1.linear.weight', tensor([[-0.0100, -1.2624, 0.0729, 0.1563, -0.8477, -1.0879, 0.4335, -0.7080,

-0.5330, 0.4685, 0.9812, -0.5562, 0.3997, 0.4231, 0.4197, -0.1196],

[-0.4007,   0.1751, -0.4889, -0.0121,   0.9361,   0.4418, -0.6923,   0.8902,
-0.1091, -0.2062, -0.6073, -0.1838, -0.3949, -0.9190, -0.9112, -0.7310]])), ('net.1.linear.bias', tensor([0.6321, 0.4978])), ('net.1.batch_norm.weight', tensor([0.9434, 1.0952])), ('net.1.batch_norm.bias', tensor([-0.2401,   0.1441])), ('net.1.batch_norm.running_mean', tensor([2.0433, 3.2356])), ('net.1.batch_norm.running_var', tensor([13.2826,   9.6024])), ('net.1.batch_norm.num_batches_tracked', tensor(144)), ('net.2.weight', tensor([[-0.4560,   0.6585]]))])

PFS model

OrderedDict([('net.0.linear.weight', tensor([[8.2368e-01, -1.4884e+00, 6.6771e-02, 2.9189e-01, -1.4935e+00,
1.8379e+00, -5.1961e-01, -6.0786e-02, 1.1468e+00, -4.5244e+00,
-4.1782e+00, -4.4847e+00, -3.0970e-01, 7.3052e-01, 2.4486e+00,
2.3629e+00, -3.9536e+00, -2.6396e-02, 2.1575e+00, 1.7702e+00,
-1.1525e+00, -1.4191e-01, 1.9272e+00, 1.8352e-01, 5.9450e-01,
-2.4458e+00, -1.1602e+00, -5.5325e+00, 1.1037e+00, -1.0336e+00,
4.3916e-01, -8.2325e-01, 2.8023e-01, 2.1396e-01, 1.2942e+00,
-1.3999e-03, 1.0358e-01, -2.0064e-01, 1.7367e-01, -6.7549e-01,
4.2942e-01, 1.1332e+00, -2.2243e+00, -1.3290e+00, 2.8954e+00,
3.7742e-01, -2.4595e-01, 4.9514e-01, -8.2180e-01, 5.2969e-01,
-2.8754e-01, 9.2541e-01, -2.0755e+00, 2.9479e+00, 2.9733e-01,
-5.2079e-01],
[1.4308e+00, 8.2931e-01, -6.9454e-01, 1.3270e+00, -6.6884e-02,
5.3856e-01, -6.0517e-01, -2.2033e-01, 8.3651e-01, 2.0932e+00,
5.8403e+00, 1.6397e+00, -1.6170e-01, -5.2818e-01, -1.2561e+00,
5.6751e-01, 2.7725e+00, -7.0042e-01, 1.4529e-01, 6.3063e-01,
1.4352e+00, -2.9802e+00, 2.1851e+00, -3.7778e+00, -2.9324e+00,
-2.4524e-01, 3.0331e+00, 3.1361e+00, -2.9677e+00, -9.9592e-01,
-6.5110e-01, 1.0572e+00, 2.8657e-02, -1.0573e+00, 1.1513e+00,
1.6808e+00, -2.8440e+00, 4.1952e-01, 3.0980e-01, 1.2544e+00,
-9.9786e-01, 4.9869e-01, 6.5395e-01, 6.5261e-01, -1.1523e-01,
4.7522e-01, -2.8780e-01, 1.1431e+00, -1.4957e+00, -1.5788e+00,
-6.3077e-02, 4.7765e+00, 4.8301e-01, -2.6440e-01, 1.6911e+00,
-1.3437e+00],
[1.4543e+00, -1.1446e+00, -4.4874e-01, 3.8274e-01, -1.6068e+00,
1.7019e+00, 7.9284e-01, -3.1145e-01, 4.2009e-01, 3.2218e+00,
-9.2278e-01, -1.5522e+00, -3.3853e-01, 2.1391e-01, 1.8360e+00,
4.7284e-01, -2.4566e+00, -9.3170e-01, 8.5096e-01, 2.5510e-01,
1.4612e-01, -1.4472e+00, 1.9705e+00, -1.3762e+00, -1.3108e+00,

-9.6548e-01, 5.1834e-01, -1.3445e+00, 5.1862e-01, -1.2678e+00,
-2.9987e-01, -4.0585e-01, 6.6603e-01, -1.7225e+00, 1.0036e+00,
5.4350e-01, -5.9928e-01, 5.1074e-01, -3.7462e-01, -4.7825e-01,
1.4996e-01, 7.1320e-01, -1.1038e+00, -1.1793e+00, 2.3737e+00,
1.2870e+00, -1.4584e+00, 5.6624e-01, -5.8250e-01, 5.7912e-01,
-3.9615e-01, 2.5305e+00, -8.7643e-01, 1.0160e+00, 8.7589e-01,
-6.5903e-01],
[-2.7512e-01, -7.3792e-01, -4.6270e-01, 6.2838e-01, 1.3321e+00,
-6.3484e-01, 2.5078e-01, -1.6337e-01, -1.5810e-01, 1.2048e-01,
2.6736e+00, 1.4258e+00, 1.3456e+00, 4.6028e-01, -2.1851e+00,
-9.1920e-01, 4.0453e+00, 1.5385e-01, -7.7997e-01, 5.1921e-02,
-2.5354e-01, 8.7256e-01, -7.6109e-02, 9.0946e-01, -3.3928e-01,
7.9255e-01, -1.7871e-02, 1.7939e+00, -1.8269e+00, 8.3167e-01,
-1.9613e+00, 2.6253e+00, -1.6847e-01, -1.4608e+00, 6.0198e-02,
-7.2619e-01, 1.7988e+00, 1.9723e-01, 3.1559e-01, 8.4483e-01,
-6.4045e-01, -4.1850e-01, 1.4156e+00, 3.7009e-01, -3.3117e-01,
-4.7375e-01, 8.2272e-01, 3.7003e-01, -3.3937e-01, 4.0384e-03,
1.0697e+00, -3.3945e+00, 1.6394e+00, -3.9901e+00, -1.1820e+00,
2.2387e+00],
[1.2814e+00, -2.5043e-01, 3.6608e-01, 1.7164e-01, -1.1051e+00,
9.5119e-01, 2.5450e-01, 8.4230e-02, -3.4605e-01, -5.2042e-01,
-4.5881e-01, -6.6795e-01, -2.8524e-02, 4.9042e-02, 5.7178e-01,
1.3062e+00, -1.7643e+00, 2.8344e-01, 1.5581e+00, 9.7976e-01,
-1.0233e+00, -4.7698e-01, -1.2125e+00, 7.6285e-01, 2.1437e+00,
3.7880e-02, -9.8579e-02, 4.0477e-03, 6.6674e-02, -1.1656e+00,
7.9795e-01, -7.8692e-01, 7.5645e-01, 5.5108e-01, 4.8307e-01,
-1.8676e-01, 1.4771e+00, 9.0480e-01, -2.4670e-01, 8.1247e-02,
4.2123e-01, 1.5886e+00, -1.0302e+00, 3.0764e-01, 5.6979e-01,
5.7251e-01, 1.9990e-01, 1.0894e+00, -3.2935e-01, 1.2591e+00,
-3.0243e-01, -9.5232e-01, -1.3494e-01, 1.0263e+00, 7.2637e-01,

-3.9218e-01],
[8.2016e-01, -2.6979e-02, -4.1070e-01, 1.1081e+00, 4.0997e-01,
5.0790e-01, 1.4552e+00, -2.2860e-01, -1.8841e-01, 3.9365e-01,
5.9620e+00, 1.8636e+00, -1.1600e+00, -1.6088e+00, -1.8350e+00,
1.5200e+00, 3.4674e+00, 1.4705e-01, -9.8553e-01, -4.3097e-01,
5.9148e-01, 5.9032e-01, 1.0215e+00, -2.4391e-01, -1.7009e+00,
2.6084e+00, -1.9674e-01, 4.5914e+00, -2.0210e+00, 9.6177e-01,
-2.4779e+00, 1.1892e+00, 3.6087e-01, -3.2296e+00, 1.3685e+00,
-1.4683e-01, 1.8034e+00, 1.3501e+00, -3.0986e-01, 1.1222e+00,
-6.9437e-01, 2.9852e-01, 2.0936e-01, 3.4988e-01, -1.4289e-01,
-1.1932e+00, 1.6875e+00, 1.3535e+00, -1.0585e+00, -4.5258e-01,
8.4100e-01, -1.9698e+00, 6.4154e-01, -9.6561e-01, -1.5634e+00,
3.5243e+00],
[2.9958e-01, -3.3353e-01, 7.7974e-01, -6.9057e-01, -5.6297e-01,
-2.1211e-01, 4.7544e-01, -6.1757e-01, -4.7124e-01, 2.0953e-01,
-2.8179e-01, 6.3345e-01, -3.3962e-01, -2.9897e-01, 7.0483e-02,
3.2374e-01, 4.7348e-02, 1.1296e-01, 2.3555e-01, -3.1963e-01,
-3.2845e-01, 2.5402e-01, -1.2561e-01, 1.8400e-01, 3.7157e-01,
5.3609e-01, 5.1927e-01, 5.7478e-01, -4.2816e-01, 4.9948e-02,
-2.0770e-02, -4.8210e-01, -3.1205e-01, 3.6648e-01, -1.3652e-01,
-6.2539e-01, 1.5520e-01, -4.1294e-01, -4.8669e-01, -8.1809e-01,
3.6625e-01, -2.5453e-01, 9.6747e-02, -3.8278e-01, 2.9768e-01,
-5.3165e-01, 5.2099e-01, -5.7780e-01, 5.1412e-02, -4.6722e-01,
-4.0666e-01, 1.6911e-01, -3.6026e-01, -2.6066e-01, -7.6353e-02,
-3.9805e-01],
[2.3411e-01, -2.0173e-01, 7.0878e-02, -2.5299e-01, -2.4269e-01,
-3.4772e-01, -2.1765e-01, -2.2485e-01, 8.0473e-02, -2.3954e-01,
8.5406e-02, 2.0977e-01, -3.9777e-01, -5.3967e-02, -2.8230e-01,
-1.7243e-01, -1.8163e-01, -2.8699e-01, 3.6114e-01, -4.0649e-01,
-7.6016e-01, 1.7628e-01, -2.5623e-01, 9.3937e-02, 2.8543e-01,

-3.4810e-01, -3.9123e-01, 3.8349e-01, 5.8824e-02, 1.2652e-01,
-3.9888e-01, -2.5266e-01, -1.8296e-01, -1.7372e-01, -2.4323e-01,
-6.1724e-01, 5.1781e-02, -3.1722e-01, -7.2084e-02, -3.9200e-01,
-6.5185e-01, -2.3809e-01, -4.3225e-01, -2.8712e-02, -3.0055e-01,
-4.2722e-01, -4.0116e-01, -6.4771e-01, -2.5053e-01, -2.7479e-01,
1.5796e-01, -4.3983e-01, -2.4293e-02, 1.3216e-01, -3.8696e-01,
-7.6045e-02]]])), ('net.0.linear.bias', tensor([0.1959, 0.4151, 0.0957, 0.2856, 0.8959, 0.7813, -0.5699, -0.2168])), ('net.0.batch_norm.weight', tensor([2.4443, 2.5621, 2.0125, 1.3650, -0.3136, 1.6840, 2.2850, 1.3003])), ('net.0.batch_norm.bias', tensor([1.3180, -0.9879, 1.5028, -0.0322, 0.6565, 0.5181, 0.6367, 0.3400])), ('net.0.batch_norm.running_mean', tensor([4.0366e+00, 9.4098e+00, 4.8445e+00, 2.5298e+00, 4.5993e+00, 7.8524e+00,
2.3556e-03, 2.7798e-37])), ('net.0.batch_norm.running_var', tensor([1.1815e+01, 2.2573e+01, 1.0513e+01, 5.7688e+00, 4.3745e+00, 1.2718e+01,
1.7358e-03, 9.0586e-35])), ('net.0.batch_norm.num_batches_tracked', tensor(744)), ('net.1.linear.weight', tensor([[-1.3820, -0.4976, -2.2852, 0.7406, 0.1955, 0.0365, 0.7902, -0.8971],
[0.8189, 0.0711, 0.1210, -1.7927, -0.5512, -3.9819, -0.7800, -0.0896],
[-0.7781, 2.9727, 0.9242, 0.0482, -0.3331, 0.7064, 0.5326, 0.2681],
[0.0661, 2.2304, 0.0839, 0.7472, 0.9619, 0.0414, 1.6486, 1.0531],
[-0.7058, 3.4245, 0.9350, 0.3568, 0.3915, 0.6109, 1.3510, -0.5249],
[-3.5893, 0.2319, -0.3384, 1.2563, 0.3278, 0.0481, 0.6002, 2.0709],
[-2.0628, -0.3070, -2.3384, 0.9127, 0.6386, -0.1309, 1.4569, -1.8342],
[-2.0549, -0.7239, -4.4490, 0.5055, -0.1009, -0.0101, 0.2813, 0.1968]])), ('net.1.linear.bias', tensor([-0.7316, 1.5140, 1.0845, 1.0298, 1.8451, 1.3233, -0.1293, -0.0910])), ('net.1.batch_norm.weight', tensor([0.6444, 0.5777, 0.4452, 0.5903, -0.6077, 0.6056, 0.8610, 0.8873])), ('net.1.batch_norm.bias', tensor([-0.0500, -0.1117, 0.0476, -0.0799, -0.0929, 0.2126, -0.3328, 0.2054])), ('net.1.batch_norm.running_mean', tensor([2.0871, 3.7600, 3.8762, 3.1479, 4.9746, 3.3235, 2.5377, 2.9539])), ('net.1.batch_norm.running_var', tensor([14.0797, 35.6703, 32.1241, 15.2261, 43.0289, 23.6371, 19.7715, 29.5523])), ('net.1.batch_

norm.num_batches_tracked', tensor(744)), ('net.2.weight', tensor([[-0.2340, 0.2458, -0.2094, -0.2573, 0.1755, -0.2871, -0.5102, -0.4274]]))])

OS model

OrderedDict([('net.0.linear.weight', tensor([[6.4050e-02, -8.9133e-01, 2.4115e-01, 2.6529e-02, -2.7966e-01,
-3.4504e-01, 9.4347e-02, -5.5284e-01, -2.3517e-01, 5.2012e-01,
-3.0080e-01, 4.5473e-03, 8.1099e-02, -1.2197e-01, -2.1331e-01,
8.3545e-01, -7.5931e-01, 7.5949e-02, -2.8750e-01],
[-5.3969e-02, -5.7556e-01, -6.2311e-01, -1.9280e-01, -7.9142e-01,
-3.6684e-02, -8.5780e-01, -2.1831e-01, 8.5640e-02, -8.2137e-03,
-4.6390e-01, -2.5827e-01, -2.9124e-01, 2.7867e-01, 9.5857e-01,
-2.1007e-01, 1.1465e-01, -2.4074e-01, 2.4973e-01],
[-1.5033e+00, 1.2500e-01, 1.6579e+00, -7.9806e-02, 9.1997e-02,
-8.6520e-02, -2.8950e-01, -3.9327e-01, 6.3778e-01, 1.3856e+00,
-2.1236e+00, -7.1156e-01, 3.4596e-01, -1.7763e+00, 3.5870e-01,
2.0344e+00, 9.0917e-01, 1.4709e+00, -1.5686e+00],
[1.0342e-01, -4.6443e-01, -7.5505e-02, 1.6734e-01, -1.1418e+00,
-2.5201e-01, -6.2679e-01, -1.6286e-02, -3.4232e-01, 8.7880e-01,
-6.5688e-01, -3.5687e-01, -4.3871e-01, -5.2154e-01, -1.4501e-01,
2.3418e-01, 2.7387e-01, -5.2524e-01, -8.2365e-03],
[5.1857e-01, 8.1806e-01, -4.6807e-01, 2.0836e+00, 2.2740e-01,
3.9199e-01, 6.8624e-01, 2.7419e-01, 7.0499e-01, -1.8734e+00,
8.6305e-01, -4.0974e-01, -5.7189e-02, -3.0818e-02, 5.7579e-01,
-1.0555e+00, 1.9328e-01, -1.6525e+00, 1.3698e+00],
[8.9447e-01, -3.6256e-01, -1.9989e-01, -8.3089e-01, 1.0902e-01,
-5.2698e-01, -1.1571e-01, -4.0207e-01, -1.2858e-01, 1.2817e-01,
-9.1524e-02, -2.6980e-01, 5.3977e-01, -2.9890e-01, -1.7893e-01,
-2.2386e-01, 5.9788e-02, -3.2357e-01, -2.4294e-01],
[-3.3937e-01, -1.3502e-01, -4.0378e-01, -4.5630e-01, 2.2178e-01,
-1.0460e-01, -7.0781e-02, -7.3206e-01, -5.3605e-01, 5.5388e-01,
-8.4109e-01, 2.3607e-01, -2.0486e-01, 2.2928e-01, -1.7943e-03,
6.0697e-01, -6.0418e-01, -1.0408e-01, -8.0523e-01],
[6.7489e-01, -5.1987e-02, 5.0473e-01, -1.6593e-01, 8.2696e-01,

-1.9482e-01, 3.3188e-01, -1.7337e-02, -2.7843e-01, 1.8007e-01,
-1.5873e-01, -8.8828e-02, 5.7400e-01, 3.1716e-01, 1.1146e+00,
6.8290e-01, 4.8619e-01, 1.4232e+00, 1.5249e-01],
[-2.0807e-01, 7.4926e-01, 1.0776e+00, 1.1687e+00, -7.3465e-02,
3.0252e-02, 5.3547e-01, -5.3082e-03, 1.2352e+00, -1.4554e+00,
8.9259e-01, -5.4559e-01, -5.2708e-01, -3.7846e-01, 8.2328e-01,
-5.6283e-01, 8.8984e-01, 1.2673e-01, -4.8220e-01],
[1.1221e-01, -7.5108e-01, 6.5012e-01, 1.1699e-01, 8.0554e-01,
-6.2109e-02, 8.3845e-01, -5.5665e-01, 5.8405e-01, 1.8565e+00,
-2.8970e+00, -4.3403e-01, -3.6024e-01, -8.2723e-01, -1.0748e+00,
4.1993e-02, -5.2504e-01, 3.9367e-01, -3.6132e+00],
[2.1092e-01, -2.5361e-01, -8.1492e-01, -8.2219e-01, -5.8135e-01,
1.4054e-01, -4.3898e-01, -2.3455e-01, -8.4715e-01, -3.4253e-01,
-1.7997e-01, 7.6053e-03, -3.7810e-02, 1.7424e-01, 1.7538e-01,
-1.1141e-01, -2.9220e-02, -5.3950e-01, 5.2390e-01],
[2.6669e-01, 4.8354e-01, 4.3170e-01, 1.1091e+00, 5.6924e-02,
2.7579e-01, 1.1920e+00, 7.6312e-01, 3.8770e-01, -1.4630e+00,
1.4292e+00, 6.4597e-01, -8.8950e-01, 5.2424e-01, -2.3144e-01,
-3.0199e-01, 1.8959e+00, -1.8227e+00, -9.1954e-01],
[6.9869e-01, -1.5741e-01, 4.2078e-02, 7.7653e-01, 9.9631e-02,
4.2618e-01, 3.3626e-01, 2.3202e-01, 4.6778e-01, -1.4427e-01,
1.8359e+00, 5.3846e-01, -6.9539e-01, 1.0974e+00, 4.0514e-01,
-1.6690e-01, -2.5068e-01, -1.7447e+00, 1.2195e+00],
[4.5512e-03, -6.4363e-02, 4.7509e-02, -6.5709e-01, -4.3240e-01,
-2.6763e-01, 2.8041e-01, -1.1404e+00, -7.6291e-01, 1.6181e-01,
-1.6710e-01, -1.1192e-02, 2.3861e-01, 4.5291e-01, 1.1126e-01,
-2.5764e-01, -7.0517e-01, -4.7132e-01, 1.8114e-01],
[3.1707e-01, 5.0009e-01, -1.4901e-01, 4.9182e-01, -1.4711e-01,
-1.0060e-01, -3.8176e-01, -6.4755e-01, -3.1935e-01, -4.0202e-01,
3.8733e-01, -2.3657e-01, 6.3308e-01, -4.3931e-01, -2.1513e-02,

-4.5427e-02, -6.5603e-01, -1.0498e-01, 1.3838e-01],

[7.9882e-01, -1.8566e-01, -5.5232e-01, -5.8008e-01, 3.6833e-01,

3.5476e-01, 7.1937e-01, -6.4457e-01, -9.7708e-02, -1.0986e+00,

1.5774e+00, -9.6316e-01, -4.5074e-01, 3.6168e-01, 2.9405e-01,

3.8137e-01, 1.7267e+00, -2.5796e+00, -3.3867e+00]])), ('net.0.linear.bias', tensor([-0.1843, -0.1701, 1.3370, -0.2278, -0.4302, -0.2862, -0.4380, 0.3943,

0.4923, 0.3752, -0.4949, 0.0784, 0.1628, -0.2828, 0.1275, -0.3643])), ('net.0.batch_norm.weight', tensor([0.9290, -0.0475, 1.8510, 1.8084, 1.4909, 0.8055, 0.7638, 0.9312,

1.3933, 1.5602, 1.3003, 1.9151, 1.3595, 0.6997, 1.0000, 1.3843])), ('net.0.batch_norm.bias', tensor([-0.1364, 0.2291, 0.4999, -0.3669, -0.1253, 0.0474, 0.3867, 0.2900,

-0.2957, 0.9249, 0.1711, 0.4074, -0.1261, -0.0177, -0.1808, -0.0561])), ('net.0.batch_norm.running_mean', tensor([4.8732e-03, 1.5405e-02, 4.8728e+00, 1.5728e-03, 8.7917e+00, 2.1566e-16,

3.4185e-15, 8.3623e-01, 8.6362e+00, 4.7172e+00, 6.3334e-19, 8.4784e+00,

6.4454e+00, 5.2088e-19, 0.0000e+00, 1.7134e+00])), ('net.0.batch_norm.running_var', tensor([2.7659e-03, 3.5222e-02, 4.3119e+00, 6.9288e-04, 1.0859e+01, 4.6003e-16,

2.5604e-15, 1.1362e+00, 4.5813e+00, 5.7127e+00, 2.7745e-16, 9.2298e+00,

7.7318e+00, 2.7755e-16, 2.7698e-16, 3.0311e+00])), ('net.0.batch_norm.num_batches_tracked', tensor(340)), ('net.1.linear.weight', tensor([[-0.7328, -0.2711, -1.3134, -0.4640, 0.5348, -0.4348, -0.2802, -0.4270,

0.0138, -1.2270, 0.0355, 0.0189, 0.4156, 0.3842, 0.3251, -0.8860],

[0.0153, -0.1085, 0.6835, 1.4828, 0.1836, -0.0461, -0.6221, -0.5831,

1.8858, 0.7068, -0.7638, 1.0731, -0.2589, 0.2704, 0.9540, 0.4349],

[-0.7368, -0.7438, -0.5211, -0.8866, 0.4335, 0.3683, 0.1065, 0.5455,

0.0702, -1.3829, 0.3370, 0.3321, 0.6066, 1.3924, 0.4146, 0.4432],

[-0.5034, -0.8741, -0.8583, -0.7349, 0.2513, -0.2287, -0.3577, -0.1082,

-0.0732, -1.4812, 0.7250, 0.0250, 0.2982, 0.1371, 1.6859, -0.9399],

[-0.1882, 0.3356, -1.1478, -0.1718, 1.0602, 1.1735, -0.5881, 0.2843,

-0.0177, -1.2438, 0.0176, -0.2166, 0.4028, -0.1307, 0.2893, -0.8387],
[0.1467, 0.3324, 0.2979, -0.0557, -0.8786, 0.2426, 0.3503, 0.3287,
-1.1587, -0.2541, 0.4507, -1.4622, -1.1444, 0.3373, -0.2210, -0.7270],
[0.2790, 0.2567, 0.9000, -0.9519, -0.4861, 0.1576, -0.5069, 0.2738,
-0.2080, -0.2050, -0.7049, -0.5181, -0.2801, -0.6496, -0.0619, -0.8502],
[0.6951, -0.5866, 0.9059, 0.5168, -0.5321, -0.1719, 0.3372, 0.4800,
-0.0981, 0.1650, 0.4771, -1.4414, -0.7696, 1.0490, -0.3166, -1.4156]])), ('net.1.linear.bias', tensor([0.3014, -0.4799, 0.7564, 0.6547, 0.1131, 0.8914, 0.3589, 0.7780])), ('net.1.batch_norm.weight', tensor([1.1796, 0.9363, 1.1931, 0.6641, 0.8200, 2.2205, 1.3607, 1.3734])), ('net.1.batch_norm.bias', tensor([0.7005, -0.0505, 0.0700, -0.0706, -0.0776, 0.5548, 0.2231, 0.0554])), ('net.1.batch_norm.running_mean', tensor([1.5798, 1.4916, 1.6604, 1.3601, 1.4722, 3.6908, 2.0754, 3.4195])), ('net.1.batch_norm.running_var', tensor([8.3256, 6.4409, 5.8162, 5.8076, 7.5861, 27.7884, 8.0287, 22.0635])), ('net.1.batch_norm.num_batches_tracked', tensor(340)), ('net.2.linear.weight', tensor([[0.2950, -0.8395, 0.3679, 0.3543, 0.7017, -0.8902, -0.6437, -0.4929],
[0.5086, -0.8206, 0.3052, 0.4708, 0.4870, -1.0420, 0.3356, -0.9602],
[-0.5758, -0.0771, -0.4924, -0.2160, -0.5739, 0.9165, 1.2307, 0.8655],
[1.0819, -1.1270, 0.5601, 0.6699, 0.7239, -0.8477, -0.8772, -0.8262]])), ('net.2.linear.bias', tensor([2.6091, 0.5499, 0.5328, 2.0817])), ('net.2.batch_norm.weight', tensor([0.7514, 0.3852, 1.5796, 0.8625])), ('net.2.batch_norm.bias', tensor([-0.3780, -0.0504, 0.7729, -0.3724])), ('net.2.batch_norm.running_mean', tensor([3.2118, 1.9569, 2.4703, 3.7345])), ('net.2.batch_norm.running_var', tensor([8.4551, 5.8640, 18.0043, 18.7223])), ('net.2.batch_norm.num_batches_tracked', tensor(340)), ('net.3.linear.weight', tensor([[0.2370, 0.3172, -0.5134, 0.3576],
[0.2711, 0.0470, -1.0835, 0.4974]])), ('net.3.linear.bias', tensor([2.0244, 4.0456])), ('net.3.batch_norm.weight', tensor([0.9236, 1.1002])), ('net.3.batch_norm.bias', tensor([-0.0845, -0.4023])), ('net.3.batch_norm.running_mean', tensor([1.6035, 3.2746])), ('net.3.batch_norm.running_var', tensor([0.8591, 2.6831])), ('net.3.batch_norm.num_batches_tracked', tensor(340)), ('net.4.weight', tensor([[0.5660, 0.4915]]))])
