# Supplementary material for: Assessing the efficacy of immunotherapy in lung squamous carcinoma using artificial intelligence neural network
Source: Front Immunol. 2022 Nov 28;13:1024707. doi: 10.3389/fimmu.2022.1024707 (PMC9742243; doi:10.3389/fimmu.2022.1024707)
Supplement: Supplementary file 2 [file Table_1.docx]

| Supplement Table 1. The mean and standard deviations of numerical variables of train cohort. | | |
| --- | --- | --- |
|  | Numerical variable | |
|  | Age | PS score |
| mean | 63.545455 | 1.008658 |
| standard deviations | 8.158024 | 0.279617 |
| Numerical variables subtract their means and divides by their standard deviations to standardize, and test cohort were standardized according to train cohort. | | |
