# Supplementary figures and images for: Assessing the efficacy of immunotherapy in lung squamous carcinoma using artificial intelligence neural network
Source: Front Immunol. 2022 Nov 28;13:1024707. doi: 10.3389/fimmu.2022.1024707 (PMC9742243; doi:10.3389/fimmu.2022.1024707)

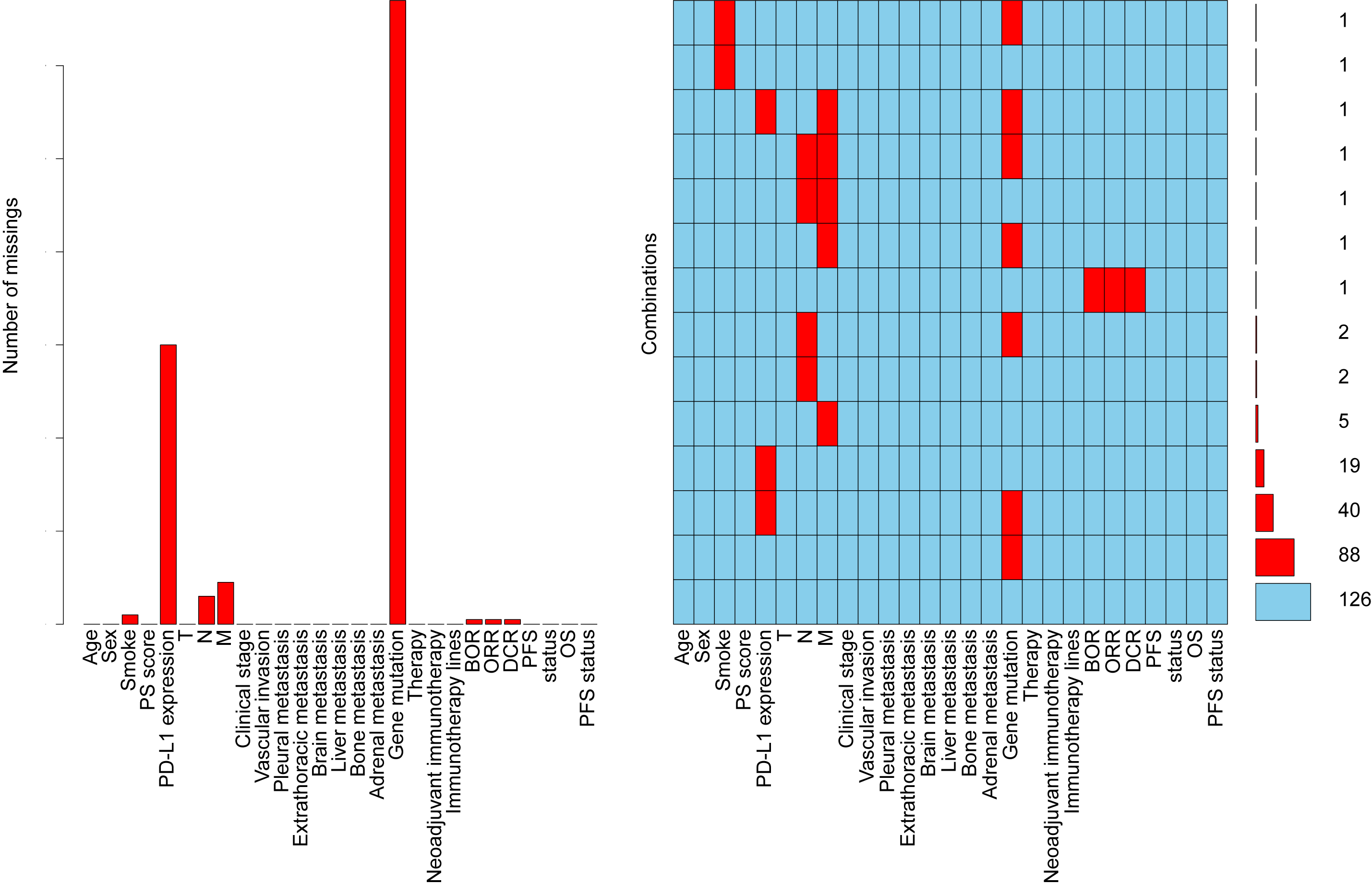

Supplement: Supplementary Figure 1 — The distribution of missing values of clinical features in this study. PD-L1, programmed cell death ligand 1. BOR, best of response. ORR, objective response rate. DCR, disease control rate. PFS, progression-free survival. OS, overall survival. [file Image_1.tif]

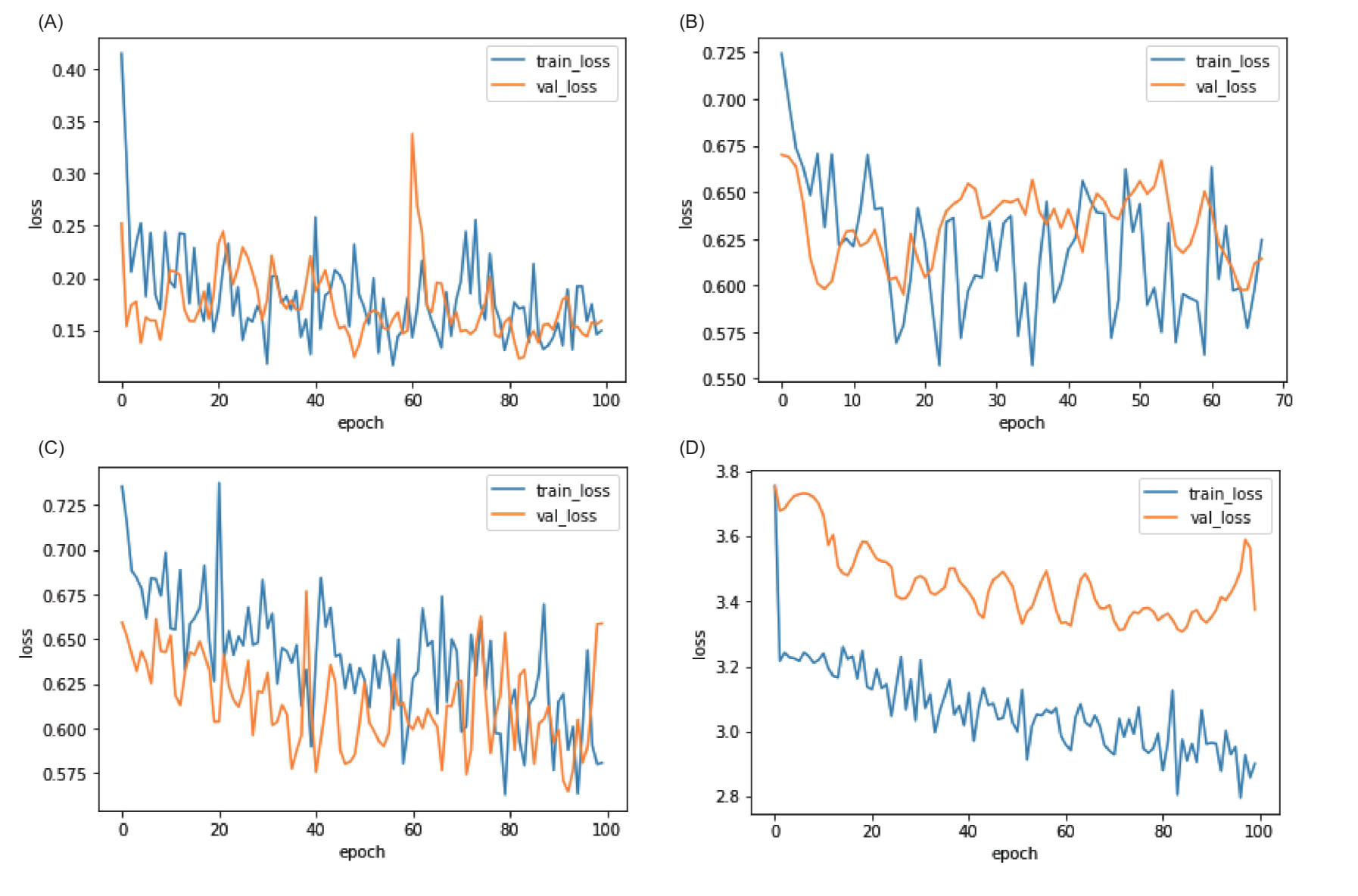

Supplement: Supplementary Figure 2 — The training curves of (A) DCR model, (B) ORR model, (C) PFS model and (D) OS model. DCR, disease control rate. ORR, objective response rate. PFS, progression-free survival. OS, overall survival. [file Image_2.tif]

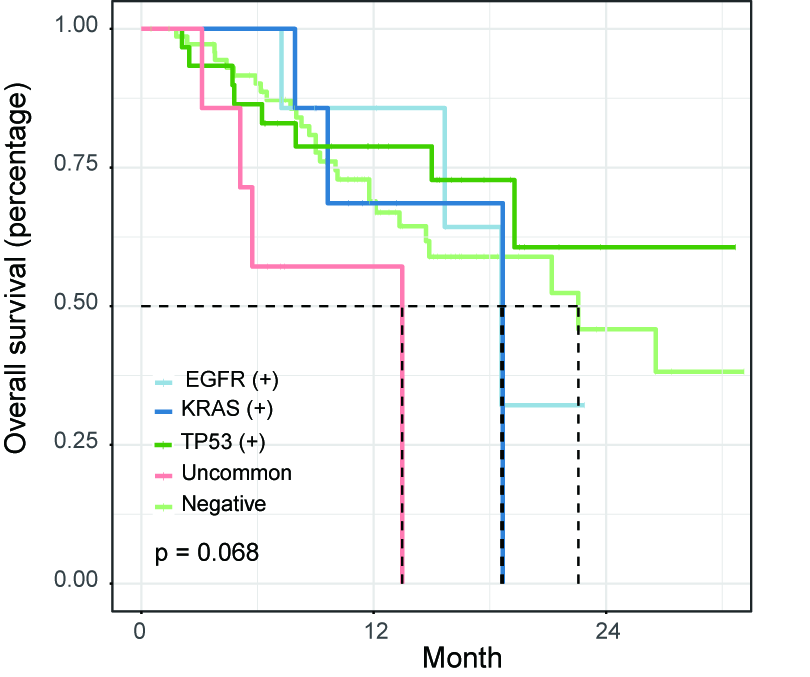

Supplement: Supplementary Figure 3 — The Kaplan-Meier curve compared the overall survival of patients with different gene mutations. Uncommon gene mutation. [file Image_3.tif]
